# A Mediterranean diet with additional extra virgin olive oil and pistachios reduces the incidence of gestational diabetes mellitus (GDM): A randomized controlled trial: The St. Carlos GDM prevention study

**DOI:** 10.1371/journal.pone.0185873

**Published:** 2017-10-19

**Authors:** Carla Assaf-Balut, Nuria García de la Torre, Alejandra Durán, Manuel Fuentes, Elena Bordiú, Laura del Valle, Cristina Familiar, Ana Ortolá, Inés Jiménez, Miguel A. Herraiz, Nuria Izquierdo, Noelia Perez, María J. Torrejon, María I. Ortega, Francisco J. Illana, Isabelle Runkle, Maria P. de Miguel, Carmen Montañez, Ana Barabash, Martín Cuesta, Miguel A. Rubio, Alfonso L. Calle-Pascual

**Affiliations:** 1 Endocrinology and Nutrition Department, Hospital Clínico Universitario San Carlos and Instituto de Investigación Sanitaria del Hospital Clínico San Carlos (IdISSC), Madrid, Spain; 2 Facultad de Medicina, Universidad Complutense de Madrid, Madrid, Spain; 3 Centro de Investigación Biomédica en Red de Diabetes y Enfermedades Metabólicas Asociadas (CIBERDEM), Madrid, Spain; 4 Preventive Medicine Department Hospital Clínico Universitario San Carlos and Instituto de Investigación Sanitaria del Hospital Clínico San Carlos (IdISSC), Madrid, Spain; 5 Gynecology and Obstetrics Department, Hospital Clínico Universitario San Carlos and Instituto de Investigación Sanitaria del Hospital Clínico San Carlos (IdISSC), Madrid, Spain; 6 Clinical Laboratory Department, Hospital Clínico Universitario San Carlos and Instituto de Investigación Sanitaria del Hospital Clínico San Carlos (IdISSC), Madrid, Spain; Weill Cornell Medical College Qatar, QATAR

## Abstract

**Background:**

Gestational diabetes mellitus (GDM) prevalence is increasing and becoming a major public health concern. Whether a Mediterranean diet can help prevent GDM in unselected pregnant women has yet to be studied.

**Methods:**

We conducted a prospective, randomized controlled trial to evaluate the incidence of GDM with two different dietary models. All consecutive normoglycemic (<92 mg/dL) pregnant women at 8–12 gestational weeks (GW) were assigned to Intervention Group (IG, n = 500): MedDiet supplemented with extra virgin olive oil (EVOO) and pistachios; or Control Group (CG, n = 500): standard diet with limited fat intake. Primary outcome was to assess the effect of the intervention on GDM incidence at 24–28 GW. Gestational weight gain (GWG), pregnancy-induced hypertension, caesarean section (CS), preterm delivery, perineal trauma, small and large for gestational age (SGA and LGA) and admissions to neonatal intensive care unit were also assessed. Analysis was by intention-to-treat.

**Results:**

A total of 874 women completed the study (440/434, CG/IG). According to nutritional questionnaires and biomarker analysis, women in the IG had a good adherence to the intervention. 177/874 women were diagnosed with GDM, 103/440 (23.4%) in CG and 74/434(17.1%) in IG, p = 0.012. The crude relative risk (RR) for GDM was 0.73 (95% CI: 0.56–0.95; p = 0.020) IG vs CG and persisted after adjusted multivariable analysis, 0.75(95% CI: 0.57–0.98; p = 0.039). IG had also significantly reduced rates of insulin-treated GDM, prematurity, GWG at 24–28 and 36–38 GW, emergency CS, perineal trauma, and SGA and LGA newborns (all p<0.05).

**Conclusions:**

An early nutritional intervention with a supplemented MedDiet reduces the incidence of GDM and improves several maternal and neonatal outcomes.

## Introduction

The prevalence of gestational diabetes mellitus (GDM) is increasing in parallel with higher rates of obesity, and older age at pregnancy [1,2]. The adoption of the International Association of the Diabetes and Pregnancy Study Groups criteria (IADPSG criteria) for its diagnosis has also increased prevalence [3]. GDM is associated with adverse maternal and neonatal outcomes and higher risk for maternal Type 2 Diabetes Mellitus (T2DM) later in life. GDM has become a major public health problem. Research into possible venues for its prevention thus becomes a priority.

Preventive strategies have been reviewed recently [4–9]. Several approaches have been studied to evaluate the effect of lifestyle interventions on the onset of GDM in high-risk women. No prevention (4–7) and prevention of GDM [8,9] results have been found, depending on the type of nutritional intervention used and the moment of its implementation.

A Mediterranean Diet (MedDiet), strengthened by the use of extra virgin olive oil (EVOO) and nuts, has been beneficial in preventing T2DM and cardiovascular disease (CVD) [10,11]. Moreover, MedDiet was retrospectively associated with a decreased risk for GDM [12]. Furthermore, adoption during the first gestational trimester of certain dietary patterns, clearly divergent from MedDiet principles, has been associated with a higher risk [13]. We have previously described an association of GDM treatment with a reduction in obesity-induced adverse pregnancy and neonatal events [14].To date, no randomized clinical trials have assessed the effect of an early nutritional intervention with MedDiet on the incidence of GDM in unselected pregnant women.

The aim of this study was to assess the effect of an intervention based on MedDiet reinforced with abundant EVOO and nuts in the form of pistachios on the incidence of GDM at 24–28 gestational weeks (GW).

## Material and methods

### Ethics statement

The study was approved by the Ethics Committee of Hospital Clínico San Carlos (full protocol approved July 17, 2013 (CI 13/296-E) and conducted according to the Helsinki Declaration. This trial was registered December 4, 2013 with the number ISRCTN84389045 (DOI 10.1186/ISRCTN84389045). The authors confirm that all ongoing and related trials for this intervention are registered.All women signed a letter of informed consent.

### Study design

This is a unicentric, clinic-based, prospective, randomized controlled trial with two parallel groups, targeting all pregnant women followed by the Obstetrics Department of the Hospital Clínico San Carlos (HCSC), Madrid, Spain. It was conducted from January 1^st^ to December 31^st^ 2015. The first woman was included in the study on January 2nd 2015 and the last one was included on December 27th 2015. The follow up until delivery finished on July 2016.

### Participants

A total of 2418 women attending their first gestational visit at 8–12 GW (Visit 0) with FBG < 92 mg/dL were assessed for inclusion. They were invited to participate upon their first ultrasound visit, between 12–14 GW (Visit 1). Gestational age at entry for inclusion was based on the one obtained in this first ultrasound. Inclusion criteria: ≥18 years old, single gestation, acceptance of participation in the study, and signature of the consent form. Exclusion criteria: gestational age at entry >14 GW, intolerance to nuts or EVOO, medical conditions or pharmacological therapy that could compromise the effect of the intervention and/or the follow-up program.

### Randomization and blinding

The randomisation and sequence allocation was performed by building a stratified randomization with permutated block-randomization, stratified by age (18–29, 30–34 and ≥35), pregestational body mass index (BMI) (<25, 25–29.9 and ≥30 kg.m^2^), parity (1 or >1), and ethnicity (Caucasian, hispanic and other), in an allocation ratio of (1:1) in blocks of 4–6.

Due to the nature of the RCT design, participants, staff and the dietician were aware of the allocation assignments. Allocation to IG/CG remained unknown to the statistician and research assistant.

### Intervention

Both the intervention group (IG) and control group (CG) were given the same basic MedDiet recommendations: ≥two servings/day of vegetables, ≥three servings/day of fruit (avoiding juices), three servings/day of skimmed dairy products, wholegrain cereals, two-three servings of legumes/week, moderate to high consumption of fish; a low consumption of red and processed meat, avoidance of refined grains, processed baked goods, pre-sliced bread, soft drinks and fresh juices, fast foods and precooked meals. They were also recommended to walk ≥30 minutes/day. These recommendations were given to women by different parties, depending on the group they were allocated to.

In one hand, participants allocated to IG received lifestyle guidance from dieticians one week after inclusion in a unique 1-hour group session. The key IG recommendation was a daily consumption of at least 40 mL of EVOO and a handful (25-30g) of pistachios. To ensure the consumption of the minimum amount recommended, women were provided at Visit 1 and 2 with 10 L of EVOO and 2 Kg of roasted pistachios each. This way, they had available 1L of EVOO and 150g of roasted pistachios weekly, throughout the pregnancy.

Women in the CG, however, were advised by midwives to restrict consumption of dietary fat, including EVOO and nuts. These recommendations are provided in local antenatal clinics as part of the available guidelines in pregnancy standard care [15].

The number of visits for the study was alike in both groups. All women were followed-up taking advantage of their scheduled standard-practice laboratory appointments. This was at first ultrasound visit (Visit 1), at 24–28 GW (Visit 2), third trimester evaluation at 36–38 GW (Visit 3) and at delivery. Nutritional guidance was reinforced at each visit for both groups. Dietary recommendations were individualized at each visit depending on GWG (according to first trimester BMI), in the context of usual recommendations. These recommendations were given in aims to reduce the caloric content of their diet when GWG exceeded the goal, by either the dietician (IG) or the midwife (CG).

### Outcomes and data collection

#### Study outcomes

The primary outcome was to compare the effect of a standard diet versus MedDiet, supplemented with EVOO and pistachios, on GDM incidence at 24–28 GW, in pregnant women with a prior normal fasting glucose (<92 mg/dL) at the first gestational visit (8–12 GW). Secondary outcomes were to assess the effect of the dietary intervention on the percent of diabetic women requiring insulin therapy, gestational weight gain (GWG), pregnancy-induced hypertension, caesarean section (CS), perineal trauma, shoulder dystocia, preterm delivery (< 37 GW), neonates SGA (small for gestational age,<10 percentile) and LGA (large for gestational age, >90 percentile) according to national charts, and admissions to the Neonatal Intensive Care Unit (NICU).

#### *GDM* screening

GDM was diagnosed at 24–28 GW with a single 2-h 75-g oral glucose tolerance test, applying IADPSG criteria. Women from both groups diagnosed with GDM were referred to the Diabetes and Pregnancy Unit and treated according to local guidelines [3]. Nutritional guidelines include daily consumption of EVOO (≥40ml/day) and nuts (a handful/day). GDM was treated with insulin and/or diet, as previously reported [3], and therapy registered. These recommendations were the same for both groups of women, regardless of belonging to the CG or IG.

One-week after diagnosis at the very latest, women had their first appointment at the Diabetes and Pregnancy Unit. To register glycemic control, women were told to perform a six-point daily glycemic profile, with fasting/preprandial and 1-h postprandial glycemias. Insulin therapy was initiated when capillary blood glucose monitoring indicated that >50% of fasting or preprandial values were >95 mg/dL(basal insulin) or 1-h postprandial levels were >140 mg/dL (bolus insulin). Insulin requirements were adjusted weekly.

#### Clinical history

A family history of T2DM and metabolic syndrome (MetS) when >2 components are present in the same relative, obstetric history of GDM and miscarriages, educational status, employment, number of prior pregnancies, smoking habit, gestational age at entry (according to 1^st^ ultrasound) were recorded at Visit 1.

#### Anthropometric data

Pregestational body weight (BW) was self-referred and registered at Visit 1. BW in each visit (1,2 and 3) was measured without shoes and with light-weight clothes. Weight gain was evaluated at 24–28 and 36–38 GW (in relation to BW at Visit 1). Blood pressure was measured with an adequate armlet when the participants had been seated for 10 minutes.

#### Biochemical variables

Blood was drawn between 08.00 and 09.00 a.m., after an overnight fast. The following data were determined: HbA1c, standardized by the International Federation of Clinical Chemistry and Laboratory Medicine (IFCC); serum insulin; HOMA-insulin resistance (HOMA-IR), calculated as glucose (mmol/L) x insulin (mcUI/ml)/22.7; and FBG. Laboratory tests were scheduled for each visit.

Urine Hydroxytyrosol levels, a biomarker of EVOO intake, was measured by liquid chromatography coupled to a Single Quadrupole LC-MS 2020 system (Shimadzu Corporation, Kyoto Japan), and serum γ-tocopherol, a biomarker of pistachio intake [16]. These biomarkers were measured at baseline and at 24–28 GW in 10% of participants randomly selected from the IG and CG.

#### Maternal outcomes

Pregnancy-induced hypertension (≥140mmHg systolic blood pressure ‒sBP‒/90 mmHg diastolic blood pressure ‒dBP‒ after 20 GW); preeclampsia(≥140mmHg systolic/90 mmHg diastolic with proteinuria ≥300 mg in 24-h after 20 GW; albuminuria (proteinuria ≥300 mg in 24-h with sBP <140 mm Hg and dBP <90 mm Hg).; urinary tract infections (UTI) (number of events requiring antibiotic treatment); and type of delivery (vaginal, instrumental or CS) and perineal trauma (any degree of spontaneous tears and episiotomy) were recorded.

#### Neonatal outcomes

Gestational age at birth, shoulder dystocia, prematurity (<37 GW), birth weight (g), height (cm) and percentiles, LGA (large for gestational age, >90 percentile), SGA (small for gestational age, <10 percentile) according to national charts, and NICU admissions were registered. Newborns of women with GDM have no specific indications of being admitted to NICU. These newborns are usually kept in Observation rooms for a 6–8 hour period, independent to the NICU unless they require NICU admission specifically for other reasons.

#### Lifestyle evaluation

At each follow-up visit, dietary intake and physical activity were evaluated. A semi-quantitative frequency questionnaire, based on the Diabetes Nutrition and Complications Trial (DNCT) study [17] and the 14-point Mediterranean Diet Adherence Screener (MEDAS) [18] were used. These were applied to questionnaires to obtain the Nutrition and MEDAS-derived PREDIMED score, respectively.

The DNCT questionnaire contains 15 items and evaluates general healthy eating habits.

Three of the items consider physical activity and 12 assess the food frequency intake.

There are three options in the questionnaire: A, B and C. Option A (value +1) is associated with DM2 prevention while option C (value -1) is associated with increased risk. Therefore, A is the most favourable habit while C is the least favourable. Option B (value 0) is the intermediate between A and C, and is the minimum objective to be achieved. The Nutrition pattern is based on twelve questions. The score is between −12 and 12, and the objective is >5.

On the other hand, the MEDAS questionnaire considers 14 items and evaluates adherence to a MedDiet. The compliance of each item provides +1 points. A score ≥ 10 is considered as ideal.

#### Sample size

For sample size calculation, the primary end-point was the incidence of GDM from 24–28 GW. Assuming a decrease of median fasting blood glucose (FBG) of -7 mg/dL following 3 months of MedDiet [19], we estimated that 315 women would be required per group to provide statistical power of 80% (2-tailed, α error of 0.05). This was calculated in order to detect a relative risk reduction of at least 30%, with a projected incidence of 35% GDM in the control group [3]. Given possible losses to follow-up and discontinuation of the intervention, we included 1000 successive women that attended their first ultrasound visit, guaranteeing a minimum duration of 12-weeks of nutritional intervention.

### Statistical analysis

Categorical variables are presented with their frequency distribution. Continuous variables are given by their mean and standard deviation (±SD). All primary analyses were performed on an intention-to-treat basis, as long as subjects reached GDM screening. Comparison between group characteristics for categorical variables was evaluated by the χ^2^ test. For continuous variables, measures were compared with Student’s t test or the Mann–Whitney U test if distribution of quantitative variables was not normal, as verified by the Shapiro-Wilk test.

The magnitude of association between the study groups and the binary outcomes was evaluated using the relative risk (RR) and 95% confidence interval (CI).

Logistic regression analyses were used to assess the effect of the intervention for the primary and secondary outcomes that were significantly different in the unadjusted model. The method proposed by Zhou [20] for estimating adjusted RR and its confidence intervals to correct the adjusted odds ratio was conducted. Crude and adjusted models were fitted for age (continuous), ethnicity and parity (model 1); for BMI (continuous) in Visit 1 (model 2); and for gestational, personal and family history, and smoker status (model 3). In the combined adjusted models, model 1 and 2 (model 4), and 1,2 and 3 (model 5) were only fitted for the primary outcome due to the small number of events in the secondary outcome variables.

All p values are 2-tailed at less than 0.05. Analyses were performed using SPSS, version 21 (SPSS, Chicago, Illinois).

## Results

**Fig 1:**
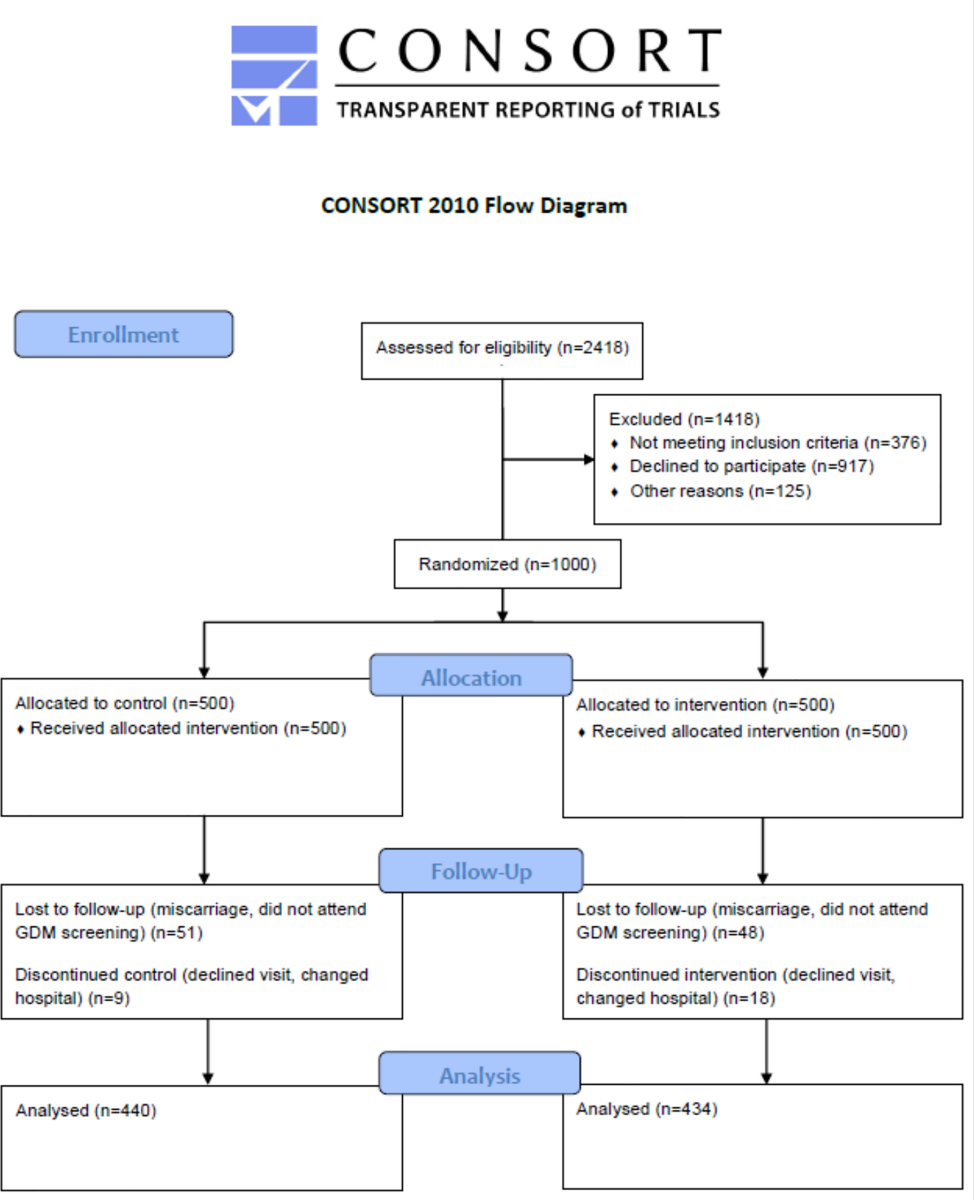
Clinical trial flow diagram.

1501/2418 women attending their first ultrasound visit were eligible and signed the consent form, of which 1000 met inclusion criteria and accepted participation (Fig 1).

They were randomly allocated to the intervention group (IG, n = 500) or the control group (CG, n = 500). Sixty women in the CG and 66 women in the IG were lost to follow-up or discontinued intervention before GDM screening. No differences were observed between them nor when compared to the women that completed the study. Consequently, 874 women were followed through postpartum discharge and analyzed. Four hundred forty were allocated to the CG and 434 to the IG (Table 1).

**Table 1:** Characteristics of the clinical trial randomized population by groups.

	CONTROL GROUP	INTERVENTION GROUP
All	N = 500	N = 500
**Age (years)**	32.7 ± 5.3	33.2 ± 5.0
**Race/Ethnicity**		
**Caucasian**	339 (67.8)	345 (69.0)
**Hispanic**	142 (28.4)	143 (28.6)
**Others**	19 (3.8)	12 (2.4)
**Family history of**		
**Type 2 Diabetes**	118 (23.6)	134 (26.8)
**MetS (>2 components)**	93 (18.6)	118 (23.6)
**Previous history of GDM**	14 (2.8)	14 (2.8)
**Previous history of miscarriages**	160 (32.2)	163(32.6)
**Educational status**		
**Elementary education**	54(10.8)	34 (6.8)
**Secondary School**	188 (37.6)	210 (42.0)
**University Degree**	251 (50.2)	252 (50.4)
**UNK**	7 (1.4)	4 (0.8)
**Employment**	376 (75.2)	390 (78.0)
**Number of pregnancies**		
**Primiparous**	211 (42.2)	232 (46.4)
**Second pregnancy**	160 (32.0)	160 (32.0)
**>2 pregnancies**	129 (25.8)	108 (21.6)
**Smoker**		
**Never**	274 (54.8)	260 (52.0)
**Current**	40 (8.0)	43 (8.6)
**Gestational Age (weeks) at baseline**	12.1 ± 0.6	12.0 ± 0.3
**Body Weight (kg)**		
**Prepregnancy**	61.7 ± 11.6	60.4 ± 10.4
**At baseline**	63.9 ± 11.9	62.5 ± 10.5
**Weigth gain**	2.1 ± 3.2	2.0 ± 2.7
**BMI (kg/m**^**2**^**)**		
**Prepregnancy**	23.3 ± 4.0	22.9 ± 3.6
**At baseline**	24.1 ± 4.1	23.7 ± 3.8
**Systolic BP(mm Hg)**	107 ± 10	107 ± 11
**Diastolic BP (mm Hg)**	65 ± 11	66 ± 9
**Fasting Blood Glucose (mg/dL)**	81.7 ± 6.1	80.0 ± 6.1
**TSH mcUI/mL**	1.9 ± 1.2	2.0 ± 1.4
**MEDAS Score**	4.9 ± 1.7	5.0 ± 1.8
**Nutrition Score**	0.5 ± 3.2	0.2 ± 3.2
**Physical Activity Score**	-1.7 ± 1.0	-1.0 ± 1.0

Data are Mean ± SD or number (%) MetS, Metabolic Syndrome. UNK, unknown. BMI, body mass index; BP, blood pressure; MEDAS Score, 14-point Mediterranean Diet Adherence Screener (MEDAS) ^18^. Nutrition Score, after Duran et al ^3^. Physical Activity Score, (Walking daily (>5 days ⁄ week) Score 0: At least 30 min. Score +1, if >60 min. Score -1, if <30 min. Climbing stairs (floors ⁄ day, >5 days a week): Score 0, Between 4 and 16; Score +1, >16; Score -1: <4)^3^

Urinary hydroxytyrosol/serum γ-tocopherol concentrations were similar in both groups at baseline. At 24–28 GW, in the IG levels increased 281 mcg/L (95% CI:193–338, p = 0.046) and 6 nmol/L (95% CI:2–123,p = 0.043), respectively, and in the CG decreased -66 mcg/L (95% CI:-158-44, p = 0.023) and -56 nmol/L (95% CI:-124-1, p = 0.036), respectively At 24–28 GW differences between both groups were p = 0.022 and p = 0.008, respectively.

Nutritional questionnaire scores and lifestyle patterns during the study are displayed in Table 2. Nutrition score and MEDAS score were similar at baseline in both groups, and significantly higher in the IG at 24–28 GW (p = 0.001) and 36–38 GW (p = 0.001).

**Table 2:** Trends in lifestyle throughout the pregnancy of the studied women.

		At baseline	24–28 GW	36–38 GW	p TREND
**EVOO (ml/day)**	Control Group	22 ± 19	26.1 ±21.1	30.1± 22.5	0.020
	Intervention Group	29 ± 23	38.3 ± 21.0	38.9 ± 25.5	0.001
	p	0.100	0.001	0.001	
**Pistacho/Nuts (day/weeks)**	Control Group	1.5 ± 2.2	1.3 ± 2.2	2.2 ± 2.9	0.128
	Intervention Group	1.3 ± 2.0	3.9 ± 2.7	3.4 ± 2.7	0.001
	p	0.146	0.001	0.001	
**Nutrition Score**	Control Group	0.5 ± 3.2	1.1 ± 3.6	3.4 ± 3.7	0.001
	Intervention Group	0.4 ± 3.2	4.2 ± 3.2	6.2 ± 3.5	0.001
	p	0.113	0.001	0.001	
**Med Diet Score**	Control Group	4.84 ± 1.74	5.81 ± 1.62	6.66 ± 1.77	0.001
	Intervention Group	4.95 ± 1.66	7.50 ± 1.48	7.81 ± 1.89	0.001
	p	0.401	0.001	0.001	
**Physical Activity ≥0 N (%)**	Control Group	58 (13.2)	29 (6.6)	17 (3.8)	0.001
	Intervention Group	41 (9.4)	29 (6.7)	22 (5.1)	0.001
	p	0.170	0.211	0.098	

Data are mean ± SD or n (%)

EVOO, extra virgen olive oil. MEDAS Score, 14-point Mediterranean Diet Adherence Screener (MEDAS) ^18^. Physical Activity Score ≥0, (Walking daily (>5 days ⁄ week) Score 0: At least 30 min. Score +1, if >60 min. Score -1, if <30 min. Climbing stairs (floors ⁄ day, >5 days a week): Score 0, Between 4 and 16; Score +1, >16; Score -1: <4)^3^

p, denote differences between groups each time (T-test) and each group compared to baseline for trend (ANOVA).

Table 3 shows information on maternal and neonatal outcomes. 177 women were diagnosed with GDM, 103/440 (23.4%) women in the CG and 74/434 (17.1%) in the IG, (p = 0.012). Compared to the CG, IG had significantly lower levels of FBG (p = 0.001), 2h post glucose load (p = 0.042), HbA1c levels (p = 0.001) and HOMA-IR (p = 0.045) at 24–28 GW. IG FBG and HbA1c levels remained significantly lower at 36–38 GW (p = 0.003 and 0.001, respectively). IG overall GWG was significantly lower at 24–28 GW and at 36–38 GW (p = 0.022 and 0.037, respectively). At 24–28 GW the GWG was significantly lower in all the three groups of women stratified by BMI (<25, 25–29.9 and ≥30 kg.m^2^). Compared to the CG, fewer women diagnosed with GDM in the IG required insulin therapy (14/74 (19%) vs. 33/103 (32%); p = 0.002), with a crude RR of 0.43 (95% CI: 0.24–0.78; p = 0.006). There was also a significant decrease in the IG in the episodes of UTI (p = 0.001), EMER-CS (p = 0.001), perineal trauma (p = 0.001) as well as a significant reduction in the rates of prematurity (p = 0.023), newborns LGA (p = 0.006) and SGA (p = 0.001), (Table 3).

**Table 3:** Maternal pregnancy and neonatal outcomes.

	CONTROL Group (N = 440)	INTERVENTION Group (n = 434)	p
***MATERNAL OUTCOMES***			
**GDM**	103 (23.4)	74 (17.1)	0.012
**75g-OGTT 24–28 GW**			
**Fasting Blood Glucose (mg/dL)**	85.7 ± 6.6	84.1 ± 6.6	0.001
**1 h Blood Glucose (mg/dL)**	123.7 ± 32.0	123.5 ± 30.2	0.912
**2 h Blood Glucose (mg/dL)**	110.0 ± 26.3	106.3 ± 23.8	0.042
**HbA1c (%) 24–28 GW**	5.1 ± 0.3	4.9 ± 0.3	0.001
**HbA1c (%) 36–38 GW**	5.3 ± 0.3	5.2 ± 0.2	0.001
**Fasting Blood Glucose 36–38 GW (mg/dL)**	77.1 ± 7.4	74 ± 7.7	0.003
**Fasting Serum Insulin (mcUI/mL)**			
**24–28 GW**	9.4 ± 5.7	9.1 ± 6.8	0.061
**36–38 GW**	10.5 ± 9.6	10.0 ± 9.9	0.085
**HOMA-IR**			
**24–28 GW**	2.2 ± 2.6	2.0 ± 1.4	0.045
**36–38 GW**	2.3 ± 2.7	2.0 ± 2.3	0.055
**Treatment of GDM**			
**Nutritional**	70 (65.4)	60 (81.0)	
**Insulin (total)**	33 (32.0)	14 (19.0)	0.037
**Bolus**	6 (5.8)	1 (1.4)	
**Basal**	23 (22.3)	12 (16.2)	
**Basal/Bolus**	4 (3.9)	1 (1.4)	0.003
**Weight gain (Kg) 12 GW to 24–28 GW**	5.6 ± 2.8	5.2 ± 2.5	0.052
**BMI**			
**<25 kg.m**^**-2**^ **(330/329)**	5.8 ± 2.7	5.4 ± 2.2	0.003
**25–29.9 (88/85)**	5.1 ± 3.0	4.7 ± 3.4	0.076
**≥ 30 (22/20)**	4.2 ± 3.5	4.0 ± 4.5	0.770
**Weight gain (Kg) 12 GW to 36–38 GW**	9.4 ± 4.3	9.9 ± 4.7	0.116
**BMI**			
**<25 kg.m**^**-2**^	9.9 ± 3.9	10.6 ± 4.0	0.096
**25–29.9**	8.8 ± 4.5	8.3 ± 6.5	0.066
**≥ 30**	5.6 ± 5.6	7.2 ± 4.7	0.583
**Systolic BP (mm Hg) 24–28 GW**	105 ± 11	105 ± 11	0.189
**Diastolic BP (mm Hg) 24–28 GW**	63 ± 9	63 ± 10	0.819
**Systolic BP (mm Hg) 36–38 GW**	112 ± 13	112 ± 11	0.193
**Diastolic BP (mm Hg) 36–38 GW**	72 ± 9	73 ± 9	0.316
**Pregnancy-induced hypertension**	19 (4.3)	13 (3.0)	0.195
**Preeclampsia**	11 (2.5)	7 (1.6)	0.247
**Albuminuria**	6 (1.4)	2 (0.5)	0.298
**Urinary Tract Infection**	60 (13.6)	24 (5.5)	0.001
**Delivery**			
**Vaginal**	312 (70.9)	316 (75.1)	
**Instrumental**	68 (15.5)	58 (11.1)	
**Cesarean section**	60 (13.6)	60 (13.8)	0.678
**Emergency**	31 (51.7)	9 (15)	0.001
**Perineal Trauma**	48 (10.9)	14 (3.2)	0.001
***NEONATAL OUTCOMES***			
**Shoulder dystocia**	1	0	N.A.
**Gestational Age at birth (weeks)**	39.6 ± 1.4	39.6 ± 1.2	0.596
**<37 GW**	17 (3.8)	5 (1.2)	0.009
**< 34 GW**	4 (0.9)	0	N.A.
**Birthweight (g)**	3215 ± 480	3250 ± 391	0.311
**Percentile**	54.5 ± 35.4	49.4 ± 27.2	0.574
**Length (cm)**	49.0 ± 2.8	49.2 ± 2.1	0.446
**Percentile**	39.0 ± 28.2	40.1 ± 28.6	0.649
**LGA >90 percentile**	18 (4.1)	4 (0.9)	0.002
**>4,500 g**	2 (0.5)	0	N.A.
**SGA <10 percentile**	25 (5.7)	5 (1.2)	0.001
**Ph Cord Blood**	7.27 ± 0.16	7.28 ± 0.07	0.714
**Ph Cord Blood ≤7**	2 (0.5)	1 (0.2)	N.A.
**Apgar Score at 1min**	8.8 ± 0.8	8.8 ± 0.7	0.658
**Apgar Score at 1min <7**	6 (1.4)	4 (0.9)	N.A.
**Apgar Score at 5 min**	10.0 ± 3.2	10.0 ± 3.	0.857
**Apgar Score at 5 min <7**	0	0	N.A.
**Hypoglycemia**	9 (2.0)	3 (0.7)	0.075
**Respiratory distress**	4 (0.9)	3 (0.7)	0.508
**Hiperbilurrubinemia**	31 (7.9)	22 (5.1)	0.140
**NICU**	14 (3.2)	8 (1.8)	0.147

GDM, Gestational Diabetes Mellitus; BP, Blood Pressure. LGA, large for gestational age. SGA, small for gestational age. NICU, Neonatal intensive care unit. NA, no available if data are not sufficient

Table 4 shows multivariable logistic regression analysis of the variables that were significantly different in the binary study. The intervention showed a crude RR for GDM of 0.73 (95%CI: 0.56–0.95; p = 0.020) and of 0.75 (95% CI: 0.57–0.98; p = 0.039) adjusted for all confounding variables.

**Table 4:** Multivariable analysis. Crude and adjusted for Adverse Outcomes Probability in the Intervention Group.

	Crude			Model 1			Model 2				Model 3		
	RR	95% CI	P	RR	95% CI	P	RR	95% CI	P		RR	95% CI	p
GDM	0.73	0.56–0.95	0.020	0.73	0.55–0.95	0.019	0.75	0.58–0.99	0.041		0.73	0.56–0.97	0.022
IT-GDM	0.43	0.24–0.78	0.006	0.44	0.25–0.81	0.008	0.51	0.28–0.93	0.028		0.43	0.24–0.78	0.005
UTI	0.40	0.25–0.63	0.001	0.41	0.26–0.64	0.001	0.41	0.26–0.65	0.001		0.41	0.26–0.64	0.001
Prematurity	0.28	0.10–0.75	0.011	0.30	0.11–0.79	0.016	0.34	0.13–0.96	0.041		0.29	0.11–0.77	0.013
SGA	0.20	0.08–0.52	0.001	0.20	0.08–0.53	0.001	0.22	0.09–0.57	0.002		0.21	0.08–0.54	0.001
LGA	0.20	0.07–0.59	0.001	0.21	0.07–0.60	0.004	0.24	0.08–0.71	0.011		0.19	0.07–0.57	0.003
EMER C-S	0.29	0.15–0.61	0.001	0.28	0.13–0.59	0.001	0.32	0.16–0.67	0.003		0.30	0.14–0.63	0.001
PT	0.21	0.12–0.36	0.001	0.20	0.11–0.35	0.001	0.21	0.12–0.37	0.001		0.21	0.12–0.36	0.001
Combined Models													
	**Model 4**			**Model 5**									
GDM	0.74	0.56–0.97	0.033	0.75	0.57–0.98	0.039							

RR, Relative Risk. 95% CI, confidence intervals.

Model 1, adjusted for age (continuous), ethnicity and parity; Model 2, adjusted for BMI (continuous); Model 3, adjusted for Gestational, and Personal and Family history, Smoker (categorical: never, current, former smoker); Model 4, Model 1 and 2; Model 5, Model 1, 2 and 3.

GDM, gestational diabetes mellitus; IT-GDM, Insulin-treated GDM; UTI, Urinary Tract Infection; SGA, Small for gestational age;LGA, large for gestational age; EMER C-S, Emergency cesarean section; PT, Perineal trauma

## Discussion

This study shows that the incidence of GDM can be reduced with an early-moderate nutritional intervention based on the supplementation of the MedDiet with an increased intake of EVOO and pistachios. Other adverse outcomes were also significantly reduced. The intervention had a significant effect on the reduction of GDM incidence considering it was applied in unselected pregnant women with a mean BMI <25kg/m^2^, considered as a low risk group. In addition, adjusted by BMI this issue remains to be significantly protective against GDM.

In previous studies, some dietary interventions improved GDM incidence and maternal/neonatal outcomes [8,9] whereas others did not [4–7]. This heterogeneity could be explained by differences in the characteristics of the study sample (high-risk women, its duration, when it was initiated, and/or the type of nutritional intervention. For instance, the UPBEAT [4] and RADIEL studies [8] were performed in women with a BMI≥30kg/m^2^, finding no reduction and reduction of GDM incidence, respectively. On the other hand, the LIMIT trial [7] was performed in women with a BMI≥25kg/m^2^, and found no reduction in GDM incidence. These sample characteristics are in contrast with the present study, that was based in an unselected sample of pregnant women with a mean BMI <25kg/m^2^. In addition, the intervention was implemented at a later stage of pregnancy [4] than that of ours and others [7,8]. Moreover, the types of intervention used were different to the present study. Some studies based their recommendations on restriction of saturated fats and consumption of carbohydrates with a low-glycemic index [4,7]. Others [8] did provide recommendations similar to ours. However, they did not provide indications of specifically increasing EVOO and nuts as well as providing them free supplies.

Therefore, none of the dietary recommendations provided in these interventions were founded in MedDiet principles supplemented with EVOO and nuts. In this respect, a single previous retrospective study found an association of adherence to a MedDiet with reduced GDM incidence [12].

The results of this study show that women in the IG gained less weight than the CG, the latter on a fat-restricted diet. A significant reduction of GDM incidence and overall GWG was also observed in other lifestyle intervention studies conducted in high-risk pregnant women [8,21–25].

Women in the IG had a lower risk for prematurity, in agreement with a recent metaanalysis [26]. UTI events and perineal trauma were also less frequent as well as were emergency CS. The UTI events were less frequent in women in the IG, in both women with NGT and GDM. EMER-CS was reduced with the intervention, mostly in women with NGT. The reduction in UTI events could be because of the association between the MedDiet with an important role in inflammation and inmunomodulation. This effect is possibly derived from the presence of food components such as phenolic compounds and oleic acid [27]. Moreover, the reduction in rates of perineal trauma could be due to improvements in the delivery progress. It could also be due to the reduction of LGA newborns since this is a risk factor of perineal trauma [28]. In the IG, the rates of LGA were significantly lower than the CG. This could explain why rates of perineal trauma were also significantly lower in the IG. It could also vouch for the lower rates in EMER CS. LGA newborns is also one of the main risk factors of EMER CS. This aspect, in turn, is associated with maternal nutrition. Specifically, LGA is inversely associated with low-glycemic index diets, which happens to be a characteristic of MedDiets.

The most relevant neonatal outcomes were the improvements in rates of SGA and LGA newborns, also found by Luoto et al [29]. SGA is a consequence of placental insufficiency. The intervention provided in this RCT could have decreased the intrauterine growth retardation, as a consequence of improved placental health [30]. In addition, Women with GDM in the IG needed less insulin treatment to achieve glycaemic goals. This means they had a lower risk of overtreatment, which could be accounted for the reduction in the rates of SGA.

Moreover, LGA is related to increased glucose transport from the mother to the fetus, and entails a greater fetal glycogen and triglyceride deposition. Thus, a reduction in maternal glycemia throughout the pregnancy would reduce fetal glycogen and triglyceride stores. Lower rates of LGA could be associated with a better glycaemic control and a lower mother-to-foetus glucose transmission. This effect could be more evident when glycemic control is achieved at an early stage of pregnancy. Therefore, the nutritional intervention reduces both extremes (SGA and LGA), without affecting the average weight of the newborn. A recent meta-analysis has also found improvements in maternal and neonatal outcomes. Insulin requirements, rates of macrosomia and hypertensive disorders were reduced following nutritional counseling [31].

As expected, the intervention used in this study achieved adherence to MedDiet as shown by the Nutrition and MEDAS-derived PREDIMED score as well as by hydroxytyrosol and γ-tocopherol levels in the IG. The MEDAS-derived PREDIMED score improved throughout the pregnancy in the IG. Yet, an ideal score of ≥10 out of 14 points was not achieved. The original questionnaire considers moderate alcohol intake and juice consumption beneficial. However, pregnant women were advised not to consume either. This suggests that the MEDAS-derived PREDIMED score might need to consider a score ≥8 as the target during pregnancy, instead of ≥10. The IG was more compliant with the MedDiet than the CG, but both seemed to improve their dietary lifestyle throughout pregnancy, particularly in the third trimester. More women in the CG developed GDM, which could explain the enhancement of this group’s scores. One possible explanation is that the nutritional recommendations given for GDM treatment are similar to the recommendations given to the IG.

Increased EVOO and pistachio consumption was clearly beneficial. EVOO is a rich source of monounsaturated fatty acids, and has been found to lower postprandial glucose levels [32] as well as to improve the inflammatory profile [33]. EVOO could have limited weight gain by reducing the carbohydrate load of meals. Furthermore, its liberal use facilitates an increased intake of vegetables, traditionally eaten with olive oil in Spanish cuisine.

Some clinical trials suggest that nuts facilitate weight loss within energy-restricted diets. It seems to be possibly due to enhanced satiety, increased thermogenesis, incomplete mastication and fat malabsorption [34,35]. Pistachios are rich in unsaturated fatty acids, fiber, magnesium, and other phytochemical constituents with potential beneficial effects on insulin sensitivity, fasting glucose levels and inflammation [34]. Their antioxidant capacity is higher than other nuts, given their high levels of lutein,β-carotene, and γ-tocopherol [35]. Pistachio consumption improves the inflammatory cytokine profiles linked to GDM development [36].

The study is not free of limitations. Most women were of Caucasian ethnicity. Therefore, our results might not be extrapolated to other populations with a different ethnical distribution. Furthermore, the observed incidence of GDM was lower than expected, reducing the statistical power for the primary outcome. According to the sample size obtained, the actual statistical power is 64%. However, the observed effect is maintained compared to that initially estimated (relative risk reduction of 30%). A higher sample size might have detected significant differences in other outcomes.

Together with its randomized design, the strengths of this study are its setting and the moderate intervention that reproduce real-life conditions. Furthermore, the follow-up throughout pregnancy permitted evaluation of maternal and fetal outcomes. Despite the low rates of overweight and obesity, the beneficial effect of the intervention persists being significant in relation to HbA1c levels. This tendency in other measured variables loses significance, probably due to sample size. In addition, when BMI is expressed as a continuous variable, all analyzed outcomes continue being significant. This might suggests that the intervention is as beneficial for overweight and obese women.

According to the data attained in this trial, limiting consumption of EVOO and pistachios is not suitable for pregnant women. Encouraging the consumption of EVOO and pistachios in a reasonable manner should be used as a tool to favour the adherence to a MedDiet. This could potentially prevent the onset of GDM and reduce its adverse outcomes.

In summary, the results show that an early dietary intervention in pregnant women with a MedDiet enriched with EVOO and pistachios reduced the incidence of GDM It also improved several pregnancy and neonatal outcomes. We would therefore recommend the adoption of this nutritional intervention by pregnant women, from the start of gestation. Important implications on the long-term health of the mother and their infant may be expected. Whether there is a beneficial impact on the future risk of T2DM, MetS, and CVDs in both women and their offspring's is being currently investigated in an ongoing study.

## Supporting information

S1 File Deindentified dataset and sintaxis. (PDF)

S2 File Original study protocol. English version.
(PDF)

S3 File Original study protocol. Spanish version.
(PDF)

S4 File DE_IDENTIFIEF_DATA SET. (SAV)

S5 File CONSORT checklist. (DOC)
