# Supplementary material for: A Mediterranean diet with additional extra virgin olive oil and pistachios reduces the incidence of gestational diabetes mellitus (GDM): A randomized controlled trial: The St. Carlos GDM prevention study
Source: PLoS One. 2017 Oct 19;12(10):e0185873. doi: 10.1371/journal.pone.0185873 (PMC5648128; doi:10.1371/journal.pone.0185873)
Supplement: S1 File — (PDF) [file pone.0185873.s001.pdf]

```
GET
  FILE='C:\Users\acall\Desktop\Repositorio\FIS NOVIEMBRE 2016 repositorio 2.sav'.
DATASET NAME Conjunto_de_datos1 WINDOW=FRONT.
FILTER OFF.
USE ALL.
EXECUTE.
T-TEST GROUPS=GRUPO_ALEATORIZADO(1 2)
  /MISSING=ANALYSIS
  /VARIABLES=EDAD Pesopregestacional PESO_SG12 BMIPREGESTACIONAL BMISG12 GananciaPesoSG12 TASSG12 TADSG12 GLUCOSA_SG12 TSHSG12 SG12_MEDDIET_SCORE
PREG_NUTRITIONSCORE
  /CRITERIA=CI(.95).
```

Prueba T

Notas

Resultados creados		31-ago-2017 18:36:53
Comentarios		
Entrada	Datos	C:\Users\acall\Desktop\Repositorio\FIS NOVIEMBRE 2016 repositorio 2.sav
	Conjunto de datos activo	Conjunto_de_datos1
	Filtro	<ninguno>
	Peso	<ninguno>
	Segmentar archivo	<ninguno>
	Núm. de filas del archivo de trabajo	1000
Tratamiento de los valores perdidos	Definición de los perdidos	Los valores perdidos definidos por el usuario serán tratados como perdidos.
	Casos utilizados	Los estadísticos de cada análisis se basan en los casos que no tienen datos perdidos ni quedan fuera de rango en cualquiera de las variables del análisis.

Sintaxis		T-TEST GROUPS=GRUPO_ALEATORIZADO(1 2)<br>/MISSING=ANALYSIS<br>/VARIABLES=EDAD Pesopregestacional PESO_SG12 BMIPREGESTACIONAL BMISG12 GananciaPesoSG12 TASSG12 TADSG12 GLUCOSA_SG12 TSHSG12 SG12_MEDDIET_SCORE PREG_NUTRITIONSCORE<br>/CRITERIA=CI(.95).
Recursos	Tiempo de procesador	00 00:00:00,031
	Tiempo transcurrido	00 00:00:00,037

[Conjunto_de_datos1] C:\Users\acall\Desktop\Repositorio\FIS NOVIEMBRE 2016 repositorio 2.sav

Estadísticos de grupo

	GRUPO_ALEATORIZADO	N	Media	Desviación típ.	Error típ. de la media
EDAD	CONTROL	500	32,73	5,254	,235
	INTERVENCION ACEITE OLIVA	499	33,19	5,019	,225
kg	CONTROL	496	61,6978	11,58256	,52007
	INTERVENCION ACEITE OLIVA	500	60,3718	10,38450	,46441
KG	CONTROL	491	63,8548	11,96589	,54001
	INTERVENCION ACEITE OLIVA	488	62,4752	10,53537	,47691
COMPUTE BMIPREGESTACIONAL = Pesopregestacional / (TALLAmetros * TALLAmetros)	CONTROL	493	23,3293	4,02408	,18124
	INTERVENCION ACEITE OLIVA	500	22,8844	3,63283	,16247
COMPUTE BMISG12 = PESO_SG12 / (TALLAmetros * TALLAmetros)	CONTROL	488	24,1461	4,13320	,18710
	INTERVENCION ACEITE OLIVA	488	23,6746	3,76139	,17027
COMPUTE GananciaPesoSG12 = PESO_SG12 - Pesopregestacional	CONTROL	488	2,1416	3,15123	,14265
	INTERVENCION ACEITE OLIVA	488	1,9713	2,68957	,12175

TASSG12	CONTROL	201	106,86	10,432	,736
	INTERVENCION ACEITE OLIVA	481	107,12	10,753	,490
TADSG12	CONTROL	201	65,31	11,330	,799
	INTERVENCION ACEITE OLIVA	481	65,73	8,608	,392
MG/DL	CONTROL	390	81,72	6,089	,308
	INTERVENCION ACEITE OLIVA	374	80,99	6,096	,315
TSHSG12	CONTROL	389	1,8759	1,18803	,06024
	INTERVENCION ACEITE OLIVA	382	2,0010	1,39213	,07123
COMPUTE SG12_MEDDIET_SCORE = SG12_ACEITEOLIVAMEDDIET + SG12_MLACEITEDIA_MEDDIET + SG12_VERDURAS_MEDDIET + SG12_FRUTAS_MEDDI...	CONTROL	497	4,8833	1,73509	,07783
	INTERVENCION ACEITE OLIVA	495	4,9758	1,75279	,07878
COMPUTE PREG_NUTRITIONSCORE = VerduraspreG + FrutapreG + AceiteolivapreG + PescadoazulpreG + PanintegralpreG + Legumbrespre...	CONTROL	499	,4910	3,24214	,14514
	INTERVENCION ACEITE OLIVA	499	,2124	3,21938	,14412

Prueba de muestras independientes

		Prueba de Levene para la igualdad de varianzas		Prueba T para la igualdad de medias						
									95% Intervalo de confianza para la diferencia	
		F	Sig.	t	gl	Sig. (bilateral)	Diferencia de medias	Error típ. de la diferencia	Inferior	Superior
EDAD	Se han asumido varianzas iguales	2,169	,141	-1,428	997	,154	-,464	,325	-1,102	,174
	No se han asumido varianzas iguales			-1,428	995,095	,153	-,464	,325	-1,102	,174
kg	Se han asumido varianzas iguales	3,346	,068	1,903	994	,057	1,32598	,69694	-,04166	2,69363

	No se han asumido varianzas iguales			1,902	980,634	,057	1,32598	,69725	-,04228	2,69425
KG	Se han asumido varianzas iguales	2,124	,145	1,914	977	,056	1,37958	,72074	-,03479	2,79396
	No se han asumido varianzas iguales			1,915	963,005	,056	1,37958	,72046	-,03427	2,79343
COMPUTE BMIPREGESTACIONAL = Pesopregestacional / (TALLAmetros * TALLAmetros)	Se han asumido varianzas iguales	2,558	,110	1,829	991	,068	,44496	,24322	-,03233	,92224
	No se han asumido varianzas iguales			1,828	977,850	,068	,44496	,24340	-,03268	,92259
COMPUTE BMISG12 = PESO_SG12 / (TALLAmetros * TALLAmetros)	Se han asumido varianzas iguales	1,707	,192	1,864	974	,063	,47144	,25298	-,02501	,96789
	No se han asumido varianzas iguales			1,864	965,472	,063	,47144	,25298	-,02501	,96790
COMPUTE GananciaPesoSG12 = PESO_SG12 - Pesopregestacional	Se han asumido varianzas iguales	1,134	,287	,908	974	,364	,17029	,18754	-,19775	,53832
	No se han asumido varianzas iguales			,908	950,541	,364	,17029	,18754	-,19776	,53833
TASSG12	Se han asumido varianzas iguales	,491	,484	-,288	680	,773	-,258	,895	-2,016	1,500
	No se han asumido varianzas iguales			-,292	385,384	,771	-,258	,884	-1,996	1,481
TADSG12	Se han asumido varianzas iguales	3,133	,077	-,534	680	,594	-,425	,797	-1,990	1,140
	No se han asumido varianzas iguales			-,478	300,815	,633	-,425	,890	-2,178	1,327
MG/DL	Se han asumido varianzas iguales	2,173	,141	1,647	762	,100	,726	,441	-,140	1,591
	No se han asumido varianzas iguales			1,647	760,589	,100	,726	,441	-,140	1,592
TSHSG12	Se han asumido varianzas iguales	2,175	,141	-1,342	769	,180	-,12502	,09315	-,30788	,05784
	No se han asumido varianzas iguales			-1,340	746,106	,181	-,12502	,09328	-,30815	,05811
COMPUTE SG12_MEDDIET_SCORE = SG12_ACEITEOLIVAMEDDIET + SG12_MLACEITEDIA_MEDDIET + SG12_VERDURAS_MEDDIET + SG12_FRUTAS_MEDDI...	Se han asumido varianzas iguales	,170	,681	-,835	990	,404	-,09246	,11074	-,30977	,12486
	No se han asumido varianzas iguales			-,835	989,801	,404	-,09246	,11074	-,30978	,12486

COMPUTE PREG_NUTRITIONSCORE = VerduraspreG + FrutapreG + AceiteolivapreG + PescadoazulpreG + PanintegralpreG + Legumbrespre...	Se han asumido varianzas iguales	,161	,688	1,362	996	,174	,27856	,20454	-,12282	,67993
	No se han asumido varianzas iguales			1,362	995,951	,174	,27856	,20454	-,12282	,67993

```
CROSSTABS
  /TABLES=ETNIA AntecedentesFamiliares ANTECGEST ESTUDIOS TRABAJO NumGestac TABAQUISMO PREG_EXERCISE_SCORE BY GRUPO_ALEATORIZADO
  /FORMAT=AVALUE TABLES
  /CELLS=COUNT COLUMN
  /COUNT ROUND CELL.
```

Tablas de contingencia

Notas

Resultados creados		31-ago-2017 18:40:54
Comentarios		
Entrada	Datos	C:\Users\acall\Desktop\Repositorio\FIS NOVIEMBRE 2016 repositorio 2.sav
	Conjunto de datos activo	Conjunto_de_datos1
	Filtro	<ninguno>
	Peso	<ninguno>
	Segmentar archivo	<ninguno>
	Núm. de filas del archivo de trabajo	1000
Tratamiento de los valores perdidos	Definición de los perdidos	Los valores perdidos definidos por el usuario serán tratados como perdidos.
	Casos utilizados	Los estadísticos de las tablas se basan en todos los casos con datos válidos en los rangos especificados para todas las variables de las tablas.

Sintaxis		CROSSTABS<br>/TABLES=ETNIA AntecedentesFamiliares ANTECGEST ESTUDIOS TRABAJO NumGestac TABAQUISMO PREG_EXERCISE_SCORE BY GRUPO_ALEATORIZADO<br>/FORMAT=AVALUE TABLES<br>/CELLS=COUNT COLUMN<br>/COUNT ROUND CELL.
Recursos	Tiempo de procesador	00 00:00:00,032
	Tiempo transcurrido	00 00:00:00,021
	Dimensiones solicitadas	2
	Casillas disponibles	174762

```
[Conjunto_de_datos1] C:\Users\acall\Desktop\Repositorio\FIS NOVIEMBRE 2016 repositorio 2.sav
```

Resumen del procesamiento de los casos

	Casos					
	Válidos		Perdidos		Total	
	N	Porcentaje	N	Porcentaje	N	Porcentaje
ETNIA * GRUPO_ALEATORIZADO	1000	100,0%	0	,0%	1000	100,0%
AntecedentesFamiliares * GRUPO_ALEATORIZADO	1000	100,0%	0	,0%	1000	100,0%
ANTECGEST * GRUPO_ALEATORIZADO	1000	100,0%	0	,0%	1000	100,0%
ESTUDIOS * GRUPO_ALEATORIZADO	1000	100,0%	0	,0%	1000	100,0%
TRABAJO * GRUPO_ALEATORIZADO	1000	100,0%	0	,0%	1000	100,0%
NumGestac * GRUPO_ALEATORIZADO	998	99,8%	2	,2%	1000	100,0%
TABAQUISMO * GRUPO_ALEATORIZADO	1000	100,0%	0	,0%	1000	100,0%

Resumen del procesamiento de los casos

	Casos					
	Válidos		Perdidos		Total	
	N	Porcentaje	N	Porcentaje	N	Porcentaje
ETNIA * GRUPO_ALEATORIZADO	1000	100,0%	0	,0%	1000	100,0%
AntecedentesFamiliares * GRUPO_ALEATORIZADO	1000	100,0%	0	,0%	1000	100,0%
ANTECGEST * GRUPO_ALEATORIZADO	1000	100,0%	0	,0%	1000	100,0%
ESTUDIOS * GRUPO_ALEATORIZADO	1000	100,0%	0	,0%	1000	100,0%
TRABAJO * GRUPO_ALEATORIZADO	1000	100,0%	0	,0%	1000	100,0%
NumGestac * GRUPO_ALEATORIZADO	998	99,8%	2	,2%	1000	100,0%
TABAQUISMO * GRUPO_ALEATORIZADO	1000	100,0%	0	,0%	1000	100,0%
COMPUTE PREG_EXERCISE_SCORE = PasearpreG + PisosescalerapreG + DeportepreG * GRUPO_ALEATORIZADO	1000	100,0%	0	,0%	1000	100,0%

Tabla de contingencia ETNIA * GRUPO_ALEATORIZADO

			GRUPO_ALEATORIZADO		
			CONTROL	INTERVENCION ACEITE OLIVA	Total
ETNIA	caucasica /incluye española	Recuento	306	326	632
		% dentro de GRUPO_ALEATORIZADO	61,2%	65,2%	63,2%
	Eslava Rumania-Bulgaria...	Recuento	33	19	52
		% dentro de GRUPO_ALEATORIZADO	6,6%	3,8%	5,2%
	Hispanica	Recuento	142	143	285
		% dentro de GRUPO_ALEATORIZADO	28,4%	28,6%	28,5%
	china	Recuento	5	0	5

		% dentro de GRUPO_ALEATORIZADO	1,0%	,0%	,5%
	africana	Recuento	10	6	16
		% dentro de GRUPO_ALEATORIZADO	2,0%	1,2%	1,6%
	otras	Recuento	3	5	8
		% dentro de GRUPO_ALEATORIZADO	,6%	1,0%	,8%
	desconocida	Recuento	1	1	2
		% dentro de GRUPO_ALEATORIZADO	,2%	,2%	,2%
Total		Recuento	500	500	1000
		% dentro de GRUPO_ALEATORIZADO	100,0%	100,0%	100,0%

Tabla de contingencia AntecedentesFamiliares * GRUPO_ALEATORIZADO

			GRUPO_ALEATORIZADO		
			CONTROL	INTERVENCION ACEITE OLIVA	Total
AntecedentesFamiliares	no existen	Recuento	156	120	276
		% dentro de GRUPO_ALEATORIZADO	31,2%	24,0%	27,6%
	diabetes	Recuento	26	16	42
		% dentro de GRUPO_ALEATORIZADO	5,2%	3,2%	4,2%
	HTA	Recuento	36	22	58
		% dentro de GRUPO_ALEATORIZADO	7,2%	4,4%	5,8%
	dislipemia	Recuento	59	49	108
		% dentro de GRUPO_ALEATORIZADO	11,8%	9,8%	10,8%
	obesidad	Recuento	37	55	92
		% dentro de GRUPO_ALEATORIZADO	7,4%	11,0%	9,2%
	2 situaciones	Recuento	92	118	210
		% dentro de GRUPO_ALEATORIZADO	18,4%	23,6%	21,0%
	mas de 2	Recuento	93	118	211

		% dentro de GRUPO_ALEATORIZADO	18,6%	23,6%	21,1%
	desconocido	Recuento	1	2	3
		% dentro de GRUPO_ALEATORIZADO	,2%	,4%	,3%
Total		Recuento	500	500	1000
		% dentro de GRUPO_ALEATORIZADO	100,0%	100,0%	100,0%

Tabla de contingencia ANTECGEST * GRUPO_ALEATORIZADO

			GRUPO_ALEATORIZADO		
			CONTROL	INTERVENCION ACEITE OLIVA	Total
ANTECGEST	ninguno	Recuento	313	322	635
		% dentro de GRUPO_ALEATORIZADO	62,6%	64,4%	63,5%
	aborto	Recuento	157	156	313
		% dentro de GRUPO_ALEATORIZADO	31,4%	31,2%	31,3%
	GDM	Recuento	12	9	21
		% dentro de GRUPO_ALEATORIZADO	2,4%	1,8%	2,1%
	HTA	Recuento	5	2	7
		% dentro de GRUPO_ALEATORIZADO	1,0%	,4%	,7%
	OTROS	Recuento	3	1	4
		% dentro de GRUPO_ALEATORIZADO	,6%	,2%	,4%
	GDM Y OTROS	Recuento	1	0	1
		% dentro de GRUPO_ALEATORIZADO	,2%	,0%	,1%
	HTA Y OTROS	Recuento	5	0	5
		% dentro de GRUPO_ALEATORIZADO	1,0%	,0%	,5%
	GDM Y aborto	Recuento	1	5	6
		% dentro de GRUPO_ALEATORIZADO	,2%	1,0%	,6%
	desconocido	Recuento	1	3	4

		% dentro de GRUPO_ALEATORIZADO	,2%	,6%	,4%
	HTA y aborto	Recuento	2	2	4
		% dentro de GRUPO_ALEATORIZADO	,4%	,4%	,4%
Total		Recuento	500	500	1000
		% dentro de GRUPO_ALEATORIZADO	100,0%	100,0%	100,0%

Tabla de contingencia ESTUDIOS * GRUPO_ALEATORIZADO

			GRUPO_ALEATORIZADO		Total
			CONTROL	INTERVENCION ACEITE OLIVA	
ESTUDIOS	SIN ESTUDIOS	Recuento	0	1	1
		% dentro de GRUPO_ALEATORIZADO	,0%	,2%	,1%
	PRIMARIOS	Recuento	54	34	88
		% dentro de GRUPO_ALEATORIZADO	10,8%	6,8%	8,8%
	BACHILLERATO	Recuento	112	120	232
		% dentro de GRUPO_ALEATORIZADO	22,4%	24,0%	23,2%
	FORMACION PROFESIONAL	Recuento	76	90	166
		% dentro de GRUPO_ALEATORIZADO	15,2%	18,0%	16,6%
	UNIVERSITARIO	Recuento	251	252	503
		% dentro de GRUPO_ALEATORIZADO	50,2%	50,4%	50,3%
	DESCONOCIDO	Recuento	7	3	10
		% dentro de GRUPO_ALEATORIZADO	1,4%	,6%	1,0%
Total		Recuento	500	500	1000
		% dentro de GRUPO_ALEATORIZADO	100,0%	100,0%	100,0%

Tabla de contingencia TRABAJO * GRUPO_ALEATORIZADO

	GRUPO_ALEATORIZADO	Total

			CONTROL	INTERVENCION ACEITE OLIVA	
TRABAJO	ESTUDIANTE	Recuento	14	10	24
		% dentro de GRUPO_ALEATORIZADO	2,8%	2,0%	2,4%
	AMA DE CASA	Recuento	10	6	16
		% dentro de GRUPO_ALEATORIZADO	2,0%	1,2%	1,6%
	FUERA DE CASA	Recuento	376	390	766
		% dentro de GRUPO_ALEATORIZADO	75,2%	78,0%	76,6%
	EN PARO	Recuento	93	91	184
		% dentro de GRUPO_ALEATORIZADO	18,6%	18,2%	18,4%
	DESCONOCIDO	Recuento	7	3	10
		% dentro de GRUPO_ALEATORIZADO	1,4%	,6%	1,0%
Total		Recuento	500	500	1000
		% dentro de GRUPO_ALEATORIZADO	100,0%	100,0%	100,0%

Tabla de contingencia NumGestac * GRUPO_ALEATORIZADO

			GRUPO_ALEATORIZADO		
			CONTROL	INTERVENCION ACEITE OLIVA	Total
NumGestac	1	Recuento	211	232	443
		% dentro de GRUPO_ALEATORIZADO	42,2%	46,6%	44,4%
	2	Recuento	160	160	320
		% dentro de GRUPO_ALEATORIZADO	32,0%	32,1%	32,1%
	3	Recuento	65	63	128
		% dentro de GRUPO_ALEATORIZADO	13,0%	12,7%	12,8%
	4	Recuento	39	26	65
		% dentro de GRUPO_ALEATORIZADO	7,8%	5,2%	6,5%
	5	Recuento	17	12	29

		% dentro de GRUPO_ALEATORIZADO	3,4%	2,4%	2,9%
	6	Recuento	6	4	10
		% dentro de GRUPO_ALEATORIZADO	1,2%	,8%	1,0%
	7	Recuento	2	0	2
		% dentro de GRUPO_ALEATORIZADO	,4%	,0%	,2%
	10	Recuento	0	1	1
		% dentro de GRUPO_ALEATORIZADO	,0%	,2%	,1%
Total		Recuento	500	498	998
		% dentro de GRUPO_ALEATORIZADO	100,0%	100,0%	100,0%

Tabla de contingencia TABAQUISMO * GRUPO_ALEATORIZADO

			GRUPO_ALEATORIZADO		Total
			CONTROL	INTERVENCION ACEITE OLIVA	
TABAQUISMO	nunca	Recuento	274	260	534
		% dentro de GRUPO_ALEATORIZADO	54,8%	52,0%	53,4%
	hasta hace 6 meses	Recuento	113	125	238
		% dentro de GRUPO_ALEATORIZADO	22,6%	25,0%	23,8%
	hasta embarazo	Recuento	69	68	137
		% dentro de GRUPO_ALEATORIZADO	13,8%	13,6%	13,7%
	fumo actualmente	Recuento	40	43	83
		% dentro de GRUPO_ALEATORIZADO	8,0%	8,6%	8,3%
	desconoce	Recuento	4	4	8
		% dentro de GRUPO_ALEATORIZADO	,8%	,8%	,8%
Total		Recuento	500	500	1000
		% dentro de GRUPO_ALEATORIZADO	100,0%	100,0%	100,0%

Tabla de contingencia COMPUTE PREG_EXERCISE_SCORE = PasearpreG + PisosescaleraspreG + DeportepreG * GRUPO_ALEATORIZADO

			GRUPO_ALEATORIZADO		Total
			CONTROL	INTERVENCION ACEITE OLIVA	
COMPUTE PREG_EXERCISE_SCORE = PasearpreG + PisosescaleraspreG + DeportepreG	-3,00	Recuento	133	149	282
		% dentro de GRUPO_ALEATORIZADO	26,6%	29,8%	28,2%
	-2,00	Recuento	180	203	383
		% dentro de GRUPO_ALEATORIZADO	36,0%	40,6%	38,3%
	-1,00	Recuento	126	101	227
		% dentro de GRUPO_ALEATORIZADO	25,2%	20,2%	22,7%
	,00	Recuento	49	38	87
		% dentro de GRUPO_ALEATORIZADO	9,8%	7,6%	8,7%
	1,00	Recuento	11	8	19
		% dentro de GRUPO_ALEATORIZADO	2,2%	1,6%	1,9%
	2,00	Recuento	1	1	2
		% dentro de GRUPO_ALEATORIZADO	,2%	,2%	,2%
Total		Recuento	500	500	1000
		% dentro de GRUPO_ALEATORIZADO	100,0%	100,0%	100,0%

```
T-TEST GROUPS=GRUPO_ALEATORIZADO(1 2)
  /MISSING=ANALYSIS
  /VARIABLES=GLUCOSABASAL75G GLUCOSA60 GLUCOSA120 HbA1_24SG HOMAIR_SG24 TAD_SG24 TAS_SG24 TAD_SG36 TAS_SG36 GLUCOSA_SG36 HbA1_SG36 HOMAIR_SG36
GANACIA_DE_PESOSG12TO24 GANACIA_DE_PESOSG12TO36 SG_PARTO PESOGR_RN TALLACM_RN PHCORDON APGAR_1MIN APGAR_5MIN
  /CRITERIA=CI(.95).
```

Prueba T

Notas

Resultados creados		02-sep-2017 09:35:48
Comentarios		
Entrada	Datos	C:\Users\acall\Desktop\Repositorio\FIS NOVIEMBRE 2016 repositorio.sav
	Conjunto de datos activo	Conjunto_de_datos1
	Filtro	DIABETESGESTACIONAL <= 2 (FILTER)
	Peso	<ninguno>
	Segmentar archivo	<ninguno>
	Núm. de filas del archivo de trabajo	874
Tratamiento de los valores perdidos	Definición de los perdidos	Los valores perdidos definidos por el usuario serán tratados como perdidos.
	Casos utilizados	Los estadísticos de cada análisis se basan en los casos que no tienen datos perdidos ni quedan fuera de rango en cualquiera de las variables del análisis.
Sintaxis		T-TEST GROUPS=GRUPO_ALEATORIZADO(1 2) /MISSING=ANALYSIS /VARIABLES=GLUCOSABASAL75G GLUCOSA60 GLUCOSA120 HbA1_24SG HOMAIR_SG24 TAD_SG24 TAS_SG24 TAD_SG36 TAS_SG36 GLUCOSA_SG36 HbA1_SG36 HOMAIR_SG36 GANACIA_DE_PESOSG12TO24 GANACIA_DE_PESOSG12TO36 SG_PARTO PESOGR_RN TALLACM_RN PHCORDON APGAR_1MIN APGAR_5MIN /CRITERIA=CI(.95).
Recursos	Tiempo de procesador	00 00:00:00,047
	Tiempo transcurrido	00 00:00:00,047

```
[Conjunto_de_datos1] C:\Users\acall\Desktop\Repositorio\FIS NOVIEMBRE 2016 repositorio.sav
```

Estadísticos de grupo

	GRUPO_ALEATORIZADO	N	Media	Desviación típ.	Error típ. de la media
SOG 75 G	CONTROL	440	85,69	6,617	,315
	INTERVENCION ACEITE OLIVA	434	84,13	6,520	,313
GLUCOSA60	CONTROL	397	123,77	31,984	1,605
	INTERVENCION ACEITE OLIVA	408	123,52	30,208	1,496
GLUCOSA120	CONTROL	396	109,93	26,326	1,323
	INTERVENCION ACEITE OLIVA	407	106,33	23,779	1,179
SOG 75 G	CONTROL	430	5,036	,2930	,0141
	INTERVENCION ACEITE OLIVA	425	4,934	,3037	,0147
HOMAIR_SG24	CONTROL	438	2,238	1,2456	,0595
	INTERVENCION ACEITE OLIVA	431	2,014	1,9320	,0931
TAD_SG24	CONTROL	357	104,66	11,001	,582
	INTERVENCION ACEITE OLIVA	422	105,71	11,101	,540
TAS_SG24	CONTROL	357	63,40	9,130	,483
	INTERVENCION ACEITE OLIVA	422	63,55	8,511	,414
TAD_SG36	CONTROL	183	111,60	13,198	,976
	INTERVENCION ACEITE OLIVA	299	112,78	11,514	,666
TAS_SG36	CONTROL	183	72,31	8,471	,626
	INTERVENCION ACEITE OLIVA	299	73,41	9,254	,535
GLUCOSA_SG36	CONTROL	191	77,09	7,352	,532
	INTERVENCION ACEITE OLIVA	314	74,99	7,767	,438
HbA1_SG36	CONTROL	189	5,322	,3366	,0245
	INTERVENCION ACEITE OLIVA	307	5,203	,3331	,0190
HOMAIR_SG36	CONTROL	179	2,211	2,6955	,2015
	INTERVENCION ACEITE OLIVA	295	2,096	2,3635	,1376
COMPUTE GANACIA_DE_PESOSG12TO24 = PESO_SG24 - PESO_SG12	CONTROL	409	5,5797	2,79643	,13827
	INTERVENCION ACEITE OLIVA	415	5,2166	2,54398	,12488

IF (PESO_SG36 > 10) GANACIA_DE_PESOSG12TO36 = PESO_SG36 - PESO_SG12	CONTROL	231	9,3645	4,32127	,28432
	INTERVENCION ACEITE OLIVA	311	9,9900	4,74947	,26932
SG_PARTO	CONTROL	364	39,5825	1,36317	,07145
	INTERVENCION ACEITE OLIVA	303	39,6368	1,25219	,07194
Peso RN en gramos	CONTROL	364	3215,8654	480,00397	25,15904
	INTERVENCION ACEITE OLIVA	305	3250,6689	391,80922	22,43493
TALLACM_RN	CONTROL	336	49,0571	2,75103	,15008
	INTERVENCION ACEITE OLIVA	284	49,2085	2,06844	,12274
PHCORDON	CONTROL	348	7,2796	,16265	,00872
	INTERVENCION ACEITE OLIVA	285	7,2758	,07646	,00453
APGAR_1MIN	CONTROL	356	8,81	,752	,040
	INTERVENCION ACEITE OLIVA	296	8,83	,706	,041
APGAR_5MIN	CONTROL	357	10,02	3,208	,170
	INTERVENCION ACEITE OLIVA	296	10,06	3,516	,204

Prueba de muestras independientes

		Prueba de Levene para la igualdad de varianzas		Prueba T para la igualdad de medias						
									95% Intervalo de confianza para la diferencia	
		F	Sig.	t	gl	Sig. (bilateral)	Diferencia de medias	Error típ. de la diferencia	Inferior	Superior
SOG 75 G	Se han asumido varianzas iguales	1,559	,212	3,504	872	,000	1,557	,444	,685	2,430
	No se han asumido varianzas iguales			3,504	871,999	,000	1,557	,444	,685	2,429
GLUCOSA60	Se han asumido varianzas iguales	1,618	,204	,111	803	,911	,244	2,192	-4,059	4,547
	No se han asumido varianzas iguales			,111	797,320	,912	,244	2,194	-4,063	4,550
GLUCOSA120	Se han asumido varianzas iguales	3,092	,079	2,033	801	,042	3,598	1,769	,124	7,071
	No se han asumido varianzas iguales			2,030	787,948	,043	3,598	1,772	,119	7,076

SOG 75 G	Se han asumido varianzas iguales	,612	,434	4,984	853	,000	,1017	,0204	,0617	,1418
	No se han asumido varianzas iguales			4,983	851,065	,000	,1017	,0204	,0617	,1418
HOMAIR_SG24	Se han asumido varianzas iguales	2,160	,142	2,031	867	,043	,2237	,1101	,0076	,4397
	No se han asumido varianzas iguales			2,025	733,043	,043	,2237	,1105	,0068	,4405
TAD_SG24	Se han asumido varianzas iguales	,253	,615	-1,314	777	,189	-1,045	,795	-2,605	,516
	No se han asumido varianzas iguales			-1,315	757,927	,189	-1,045	,794	-2,604	,515
TAS_SG24	Se han asumido varianzas iguales	,964	,326	-,228	777	,819	-,144	,633	-1,387	1,098
	No se han asumido varianzas iguales			-,227	735,626	,821	-,144	,636	-1,394	1,105
TAD_SG36	Se han asumido varianzas iguales	1,050	,306	-1,038	480	,300	-1,187	1,143	-3,433	1,059
	No se han asumido varianzas iguales			-1,005	345,308	,316	-1,187	1,181	-3,510	1,136
TAS_SG36	Se han asumido varianzas iguales	1,365	,243	-1,303	480	,193	-1,097	,841	-2,750	,557
	No se han asumido varianzas iguales			-1,331	411,028	,184	-1,097	,824	-2,716	,523
GLUCOSA_SG36	Se han asumido varianzas iguales	,176	,675	3,007	503	,003	2,101	,699	,728	3,473
	No se han asumido varianzas iguales			3,048	418,446	,002	2,101	,689	,746	3,455
HbA1_SG36	Se han asumido varianzas iguales	,063	,802	3,827	494	,000	,1183	,0309	,0576	,1791
	No se han asumido varianzas iguales			3,817	394,860	,000	,1183	,0310	,0574	,1793
HOMAIR_SG36	Se han asumido varianzas iguales	,373	,542	,487	472	,627	,1150	,2363	-,3493	,5793
	No se han asumido varianzas iguales			,471	338,254	,638	,1150	,2440	-,3649	,5949
COMPUTE GANACIA_DE_PESOSG12TO 24 = PESO_SG24 - PESO_SG12	Se han asumido varianzas iguales	5,102	,024	1,950	822	,052	,36308	,18619	-,00238	,72854
	No se han asumido varianzas iguales			1,949	812,375	,052	,36308	,18632	-,00264	,72880
IF (PESO_SG36 > 10) GANACIA_DE_PESOSG12TO	Se han asumido varianzas iguales	,001	,981	-1,575	540	,116	-,62553	,39712	-1,40562	,15455

36 = PESO_SG36 - PESO_SG12	No se han asumido varianzas iguales			-1,597	518,312	,111	-,62553	,39162	-1,39490	,14383
SG_PARTO	Se han asumido varianzas iguales	1,005	,317	-,531	665	,596	-,05427	,10218	-,25490	,14637
	No se han asumido varianzas iguales			-,535	658,554	,593	-,05427	,10139	-,25335	,14482
Peso RN en gramos	Se han asumido varianzas iguales	5,575	,019	-1,014	667	,311	-34,80347	34,31074	-102,17352	32,56659
	No se han asumido varianzas iguales			-1,032	666,558	,302	-34,80347	33,70910	-100,99228	31,38534
TALLACM_RN	Se han asumido varianzas iguales	,510	,476	-,762	618	,446	-,15131	,19846	-,54104	,23842
	No se han asumido varianzas iguales			-,780	609,974	,435	-,15131	,19388	-,53206	,22944
PHCORDON	Se han asumido varianzas iguales	,305	,581	,367	631	,714	,00384	,01047	-,01672	,02441
	No se han asumido varianzas iguales			,391	513,823	,696	,00384	,00983	-,01546	,02315
APGAR_1MIN	Se han asumido varianzas iguales	,211	,646	-,443	650	,658	-,025	,058	-,138	,088
	No se han asumido varianzas iguales			-,445	640,647	,656	-,025	,057	-,138	,087
APGAR_5MIN	Se han asumido varianzas iguales	,069	,792	-,180	651	,857	-,047	,263	-,565	,470
	No se han asumido varianzas iguales			-,178	604,207	,859	-,047	,266	-,569	,474

```
USE ALL.
COMPUTE filter_$=(DIABETESGESTACIONAL   = 1).
VARIABLE LABELS filter_$ 'DIABETESGESTACIONAL   = 1 (FILTER)'.
VALUE LABELS filter_$ 0 'Not Selected' 1 'Selected'.
FORMATS filter_$ (f1.0).
FILTER BY filter_$.
EXECUTE.
CROSSTABS
  /TABLES=TTOFINAL_INSULINAvsNUTRICION BY GRUPO_ALEATORIZADO
  /FORMAT=AVALUE TABLES
  /STATISTICS=CHISQ
  /CELLS=COUNT COLUMN
  /COUNT ROUND CELL.
```

Tablas de contingencia

Notas

Resultados creados		02-sep-2017 09:39:56
Comentarios		
Entrada	Datos	C:\Users\acall\Desktop\Repositorio\FIS NOVIEMBRE 2016 repositorio.sav
	Conjunto de datos activo	Conjunto_de_datos1
	Filtro	DIABETESGESTACIONAL = 1 (FILTER)
	Peso	<ninguno>
	Segmentar archivo	<ninguno>
	Núm. de filas del archivo de trabajo	177
Tratamiento de los valores perdidos	Definición de los perdidos	Los valores perdidos definidos por el usuario serán tratados como perdidos.
	Casos utilizados	Los estadísticos de las tablas se basan en todos los casos con datos válidos en los rangos especificados para todas las variables de las tablas.
Sintaxis		CROSSTABS<br><br>/TABLES=TTOFINAL_INSULINAvsNUTRICION BY GRUPO_ALEATORIZADO<br>/FORMAT=AVALUE TABLES<br>/STATISTICS=CHISQ<br>/CELLS=COUNT COLUMN<br>/COUNT ROUND CELL.
Recursos	Tiempo de procesador	00 00:00:00,016
	Tiempo transcurrido	00 00:00:00,016
	Dimensiones solicitadas	2
	Casillas disponibles	174762

```
[Conjunto_de_datos1] C:\Users\acall\Desktop\Repositorio\FIS NOVIEMBRE 2016 repositorio.sav
```

Resumen del procesamiento de los casos

	Casos		
	Válidos	Perdidos	Total

	N	Porcentaje	N	Porcentaje	N	Porcentaje
TTOFINAL_INSULINAvsNUTRICION * GRUPO_ALEATORIZADO	177	100,0%	0	,0%	177	100,0%

Tabla de contingencia TTOFINAL_INSULINAvsNUTRICION * GRUPO_ALEATORIZADO

			GRUPO_ALEATORIZADO		
			CONTROL	INTERVENCION ACEITE OLIVA	Total
TTOFINAL_INSULINAvsNUTRICION	1,00	Recuento	33	14	47
		% dentro de GRUPO_ALEATORIZADO	32,0%	18,9%	26,6%
	2,00	Recuento	70	60	130
		% dentro de GRUPO_ALEATORIZADO	68,0%	81,1%	73,4%
Total		Recuento	103	74	177
		% dentro de GRUPO_ALEATORIZADO	100,0%	100,0%	100,0%

Pruebas de chi-cuadrado

	Valor	gl	Sig. asintótica (bilateral)	Sig. exacta (bilateral)	Sig. exacta (unilateral)
Chi-cuadrado de Pearson	3,801[a]	1	,051		
Corrección por continuidad[b]	3,158	1	,076		
Razón de verosimilitudes	3,902	1	,048		
Estadístico exacto de Fisher				,059	,037
Asociación lineal por lineal	3,779	1	,052		
N de casos válidos	177				

a. 0 casillas (,0%) tienen una frecuencia esperada inferior a 5. La frecuencia mínima esperada es 19,65.

b. Calculado sólo para una tabla de 2x2.

```
USE ALL.
COMPUTE filter_$=(DIABETESGESTACIONAL   = 2).
VARIABLE LABELS filter_$ 'DIABETESGESTACIONAL   = 2 (FILTER)'.
VALUE LABELS filter_$ 0 'Not Selected' 1 'Selected'.
FORMATS filter_$ (f1.0).
FILTER BY filter_$.
EXECUTE.
CROSSTABS
```

```
  /TABLES=DIABETESGESTACIONAL GESTATIONAL_HTAYESvsNO HTA_EMBARAZO_PREECLAMPSIA PROTEINURIA UTIYesVSNo PARTO_NUMERICA EMERGENCY_CESAREAN_SECTION
PERINEALTRAUMAYESvsNO DAÑO_PLEXOBRAQUIAL PREMATURIDAD LGA_MACROSOMICOS SGA HIPOGLUCEMIA DISTRES_RESPIRATORIO
HPERBILIRRUNEMIA_YES_VS_NO NICU_OBSERVATION_STANCE BY GRUPO_ALEATORIZADO
  /FORMAT=AVALUE TABLES
  /STATISTICS=CHISQ
  /CELLS=COUNT COLUMN
  /COUNT ROUND CELL.
```

Tablas de contingencia

Notas

Resultados creados		02-sep-2017 10:28:59
Comentarios		
Entrada	Datos	C:\Users\acall\Desktop\Repositorio\FIS NOVIEMBRE 2016 repositorio.sav
	Conjunto de datos activo	Conjunto_de_datos1
	Filtro	DIABETESGESTACIONAL <= 2 (FILTER)
	Peso	<ninguno>
	Segmentar archivo	<ninguno>
	Núm. de filas del archivo de trabajo	874
Tratamiento de los valores perdidos	Definición de los perdidos	Los valores perdidos definidos por el usuario serán tratados como perdidos.
	Casos utilizados	Los estadísticos de las tablas se basan en todos los casos con datos válidos en los rangos especificados para todas las variables de las tablas.

Sintaxis		CROSSTABS<br>/TABLES=DIABETESGESTACIONAL GESTATIONAL_HTAYESvsNO HTA_EMBARAZO_PREECLAMPSIA PROTEINURIA UTIYesVSNo PARTO_NUMERICA EMERGENCY_CESAREAN_SECTION PERINEALTRAUMAYESvsNO DAÑO_PLEXOBRAQUIAL PREMATURIDAD LGA_MACROSOMICOS SGA HIPOGLUCEMIA DISTRES_RESPIRATORIO HPERBILIRRUNEMIA_YES_VS_NO NICU_OBSERVATION_STANCE BY GRUPO_ALEATORIZADO<br>/FORMAT=AVALUE TABLES<br>/STATISTICS=CHISQ<br>/CELLS=COUNT COLUMN<br>/COUNT ROUND CELL.
Recursos	Tiempo de procesador	00 00:00:00,032
	Tiempo transcurrido	00 00:00:00,028
	Dimensiones solicitadas	2
	Casillas disponibles	174762

[Conjunto_de_datos1] C:\Users\acall\Desktop\Repositorio\FIS NOVIEMBRE 2016 repositorio.sav

Resumen del procesamiento de los casos

	Casos					
	Válidos		Perdidos		Total	
	N	Porcentaje	N	Porcentaje	N	Porcentaje
DIABETESGESTACIONAL * GRUPO_ALEATORIZADO	874	100,0%	0	,0%	874	100,0%
GESTATIONAL_HTAYESvsNO * GRUPO_ALEATORIZADO	874	100,0%	0	,0%	874	100,0%
HTA_EMBARAZO_PREECLAMPSIA * GRUPO_ALEATORIZADO	874	100,0%	0	,0%	874	100,0%

PROTEINURIA * GRUPO_ALEATORIZADO	874	100,0%	0	,0%	874	100,0%
UTIYesVSNo * GRUPO_ALEATORIZADO	874	100,0%	0	,0%	874	100,0%
PARTO_NUMERICA * GRUPO_ALEATORIZADO	874	100,0%	0	,0%	874	100,0%
EMERGENCY_CESAREAN_SECTION * GRUPO_ALEATORIZADO	874	100,0%	0	,0%	874	100,0%
PT YES VS NO * GRUPO_ALEATORIZADO	874	100,0%	0	,0%	874	100,0%
DAÑO_PLEXOBRAQUIAL * GRUPO_ALEATORIZADO	874	100,0%	0	,0%	874	100,0%
PREMATURO <37 VS >=37 * GRUPO_ALEATORIZADO	874	100,0%	0	,0%	874	100,0%
LGA> 90 PERCENTILE * GRUPO_ALEATORIZADO	874	100,0%	0	,0%	874	100,0%
SGA < 10 PERCENTILE * GRUPO_ALEATORIZADO	874	100,0%	0	,0%	874	100,0%
HIPOGLUCEMIA * GRUPO_ALEATORIZADO	874	100,0%	0	,0%	874	100,0%
DISTRES_RESPIRATORIO * GRUPO_ALEATORIZADO	874	100,0%	0	,0%	874	100,0%
HPERBILIRRUNEMIA_YES_VS_NO * GRUPO_ALEATORIZADO	874	100,0%	0	,0%	874	100,0%
NICU_OBSERVATION_STANCE * GRUPO_ALEATORIZADO	874	100,0%	0	,0%	874	100,0%

DIABETESGESTACIONAL * GRUPO_ALEATORIZADO

Tabla de contingencia

			GRUPO_ALEATORIZADO		
			CONTROL	INTERVENCION ACEITE OLIVA	Total
DIABETESGESTACIONAL	SI	Recuento	103	74	177
		% dentro de GRUPO_ALEATORIZADO	23,4%	17,1%	20,3%

	NO	Recuento	337	360	697
		% dentro de GRUPO_ALEATORIZADO	76,6%	82,9%	79,7%
Total		Recuento	440	434	874
		% dentro de GRUPO_ALEATORIZADO	100,0%	100,0%	100,0%

Pruebas de chi-cuadrado

	Valor	gl	Sig. asintótica (bilateral)	Sig. exacta (bilateral)	Sig. exacta (unilateral)
Chi-cuadrado de Pearson	5,469[a]	1	,019		
Corrección por continuidad[b]	5,083	1	,024		
Razón de verosimilitudes	5,491	1	,019		
Estadístico exacto de Fisher				,023	,012
Asociación lineal por lineal	5,463	1	,019		
N de casos válidos	874				

a. 0 casillas (,0%) tienen una frecuencia esperada inferior a 5. La frecuencia mínima esperada es 87,89.

b. Calculado sólo para una tabla de 2x2.

GESTATIONAL_HTAYESvsNO * GRUPO_ALEATORIZADO

Tabla de contingencia

			GRUPO_ALEATORIZADO		
			CONTROL	INTERVENCION ACEITE OLIVA	Total
GESTATIONAL_HTAYESvsNO	1,00	Recuento	19	13	32
		% dentro de GRUPO_ALEATORIZADO	4,3%	3,0%	3,7%
	2,00	Recuento	421	421	842
		% dentro de GRUPO_ALEATORIZADO	95,7%	97,0%	96,3%
Total		Recuento	440	434	874
		% dentro de GRUPO_ALEATORIZADO	100,0%	100,0%	100,0%

Pruebas de chi-cuadrado

	Valor	gl	Sig. asintótica (bilateral)	Sig. exacta (bilateral)	Sig. exacta (unilateral)
Chi-cuadrado de Pearson	1,084[a]	1	,298		
Corrección por continuidad[b]	,741	1	,389		
Razón de verosimilitudes	1,090	1	,296		
Estadístico exacto de Fisher				,368	,195
Asociación lineal por lineal	1,083	1	,298		
N de casos válidos	874				

a. 0 casillas (,0%) tienen una frecuencia esperada inferior a 5. La frecuencia mínima esperada es 15,89.

b. Calculado sólo para una tabla de 2x2.

HTA_EMBARAZO_PREECLAMPSIA * GRUPO_ALEATORIZADO

Tabla de contingencia

			GRUPO_ALEATORIZADO		
			CONTROL	INTERVENCION ACEITE OLIVA	Total
HTA_EMBARAZO_PREECLAMPSIA	PREECLAMPIA	Recuento	11	7	18
		% dentro de GRUPO_ALEATORIZADO	2,5%	1,6%	2,1%
	NO	Recuento	429	427	856
		% dentro de GRUPO_ALEATORIZADO	97,5%	98,4%	97,9%
Total		Recuento	440	434	874
		% dentro de GRUPO_ALEATORIZADO	100,0%	100,0%	100,0%

Pruebas de chi-cuadrado

	Valor	gl	Sig. asintótica (bilateral)	Sig. exacta (bilateral)	Sig. exacta (unilateral)
Chi-cuadrado de Pearson	,852[a]	1	,356		

Corrección por continuidad[b]	,469	1	,493		
Razón de verosimilitudes	,860	1	,354		
Estadístico exacto de Fisher				,476	,247
Asociación lineal por lineal	,851	1	,356		
N de casos válidos	874				

a. 0 casillas (,0%) tienen una frecuencia esperada inferior a 5. La frecuencia mínima esperada es 8,94.

b. Calculado sólo para una tabla de 2x2.

PROTEINURIA * GRUPO_ALEATORIZADO

Tabla de contingencia

			GRUPO_ALEATORIZADO		
			CONTROL	INTERVENCION ACEITE OLIVA	Total
PROTEINURIA	SI	Recuento	6	2	8
		% dentro de GRUPO_ALEATORIZADO	1,4%	,5%	,9%
	NO	Recuento	434	432	866
		% dentro de GRUPO_ALEATORIZADO	98,6%	99,5%	99,1%
Total		Recuento	440	434	874
		% dentro de GRUPO_ALEATORIZADO	100,0%	100,0%	100,0%

Pruebas de chi-cuadrado

	Valor	gl	Sig. asintótica (bilateral)	Sig. exacta (bilateral)	Sig. exacta (unilateral)
Chi-cuadrado de Pearson	1,964[a]	1	,161		
Corrección por continuidad[b]	1,094	1	,296		
Razón de verosimilitudes	2,056	1	,152		
Estadístico exacto de Fisher				,287	,148
Asociación lineal por lineal	1,961	1	,161		
N de casos válidos	874				

Pruebas de chi-cuadrado

	Valor	gl	Sig. asintótica (bilateral)	Sig. exacta (bilateral)	Sig. exacta (unilateral)
Chi-cuadrado de Pearson	1,964[a]	1	,161		
Corrección por continuidad[b]	1,094	1	,296		
Razón de verosimilitudes	2,056	1	,152		
Estadístico exacto de Fisher				,287	,148
Asociación lineal por lineal	1,961	1	,161		
N de casos válidos	874				

a. 2 casillas (50,0%) tienen una frecuencia esperada inferior a 5. La frecuencia mínima esperada es 3,97.

b. Calculado sólo para una tabla de 2x2.

UTIYesVSNo * GRUPO_ALEATORIZADO

Tabla de contingencia

			GRUPO_ALEATORIZADO		Total
			CONTROL	INTERVENCION ACEITE OLIVA	
UTIYesVSNo	1,00	Recuento	60	24	84
		% dentro de GRUPO_ALEATORIZADO	13,6%	5,5%	9,6%
	2,00	Recuento	380	410	790
		% dentro de GRUPO_ALEATORIZADO	86,4%	94,5%	90,4%
Total		Recuento	440	434	874
		% dentro de GRUPO_ALEATORIZADO	100,0%	100,0%	100,0%

Pruebas de chi-cuadrado

	Valor	gl	Sig. asintótica (bilateral)	Sig. exacta (bilateral)	Sig. exacta (unilateral)
Chi-cuadrado de Pearson	16,527[a]	1	,000		
Corrección por continuidad[b]	15,607	1	,000		
Razón de verosimilitudes	17,038	1	,000		

Estadístico exacto de Fisher				,000	,000
Asociación lineal por lineal	16,508	1	,000		
N de casos válidos	874				

a. 0 casillas (,0%) tienen una frecuencia esperada inferior a 5. La frecuencia mínima esperada es 41,71.

b. Calculado sólo para una tabla de 2x2.

PARTO_NUMERICA * GRUPO_ALEATORIZADO

Tabla de contingencia

			GRUPO_ALEATORIZADO		
			CONTROL	INTERVENCION ACEITE OLIVA	Total
PARTO_NUMERICA	1,00	Recuento	312	316	628
		% dentro de GRUPO_ALEATORIZADO	70,9%	72,8%	71,9%
	2,00	Recuento	68	58	126
		% dentro de GRUPO_ALEATORIZADO	15,5%	13,4%	14,4%
	3,00	Recuento	60	60	120
		% dentro de GRUPO_ALEATORIZADO	13,6%	13,8%	13,7%
Total		Recuento	440	434	874
		% dentro de GRUPO_ALEATORIZADO	100,0%	100,0%	100,0%

Pruebas de chi-cuadrado

	Valor	gl	Sig. asintótica (bilateral)
Chi-cuadrado de Pearson	,778[a]	2	,678
Razón de verosimilitudes	,779	2	,677
Asociación lineal por lineal	,124	1	,725
N de casos válidos	874		

Pruebas de chi-cuadrado

	Valor	gl	Sig. asintótica (bilateral)
Chi-cuadrado de Pearson	,778[a]	2	,678
Razón de verosimilitudes	,779	2	,677
Asociación lineal por lineal	,124	1	,725
N de casos válidos	874		

a. 0 casillas (,0%) tienen una frecuencia esperada inferior a 5. La frecuencia mínima esperada es 59,59.

EMERGENCY_CESAREAN_SECTION * GRUPO_ALEATORIZADO

Tabla de contingencia

			GRUPO_ALEATORIZADO		Total
			CONTROL	INTERVENCION ACEITE OLIVA	
EMERGENCY_CESAREAN_SECTION	1,00	Recuento	31	9	40
		% dentro de GRUPO_ALEATORIZADO	7,0%	2,1%	4,6%
	2,00	Recuento	409	425	834
		% dentro de GRUPO_ALEATORIZADO	93,0%	97,9%	95,4%
Total		Recuento	440	434	874
		% dentro de GRUPO_ALEATORIZADO	100,0%	100,0%	100,0%

Pruebas de chi-cuadrado

	Valor	gl	Sig. asintótica (bilateral)	Sig. exacta (bilateral)	Sig. exacta (unilateral)
Chi-cuadrado de Pearson	12,366[a]	1	,000		
Corrección por continuidad[b]	11,254	1	,001		
Razón de verosimilitudes	13,064	1	,000		
Estadístico exacto de Fisher				,001	,000
Asociación lineal por lineal	12,352	1	,000		

N de casos válidos	874				

a. 0 casillas (,0%) tienen una frecuencia esperada inferior a 5. La frecuencia mínima esperada es 19,86.

b. Calculado sólo para una tabla de 2x2.

PT YES VS NO * GRUPO_ALEATORIZADO

Tabla de contingencia

			GRUPO_ALEATORIZADO		Total
			CONTROL	INTERVENCION ACEITE OLIVA	
PT YES VS NO	1,00	Recuento	68	14	82
		% dentro de GRUPO_ALEATORIZADO	15,5%	3,2%	9,4%
	2,00	Recuento	372	420	792
		% dentro de GRUPO_ALEATORIZADO	84,5%	96,8%	90,6%
Total		Recuento	440	434	874
		% dentro de GRUPO_ALEATORIZADO	100,0%	100,0%	100,0%

Pruebas de chi-cuadrado

	Valor	gl	Sig. asintótica (bilateral)	Sig. exacta (bilateral)	Sig. exacta (unilateral)
Chi-cuadrado de Pearson	38,431[a]	1	,000		
Corrección por continuidad[b]	37,006	1	,000		
Razón de verosimilitudes	41,591	1	,000		
Estadístico exacto de Fisher				,000	,000
Asociación lineal por lineal	38,387	1	,000		
N de casos válidos	874				

a. 0 casillas (,0%) tienen una frecuencia esperada inferior a 5. La frecuencia mínima esperada es 40,72.

b. Calculado sólo para una tabla de 2x2.

DAÑO_PLEXOBRAQUIAL * GRUPO_ALEATORIZADO

Tabla de contingencia

			GRUPO_ALEATORIZADO		
			CONTROL	INTERVENCION ACEITE OLIVA	Total
DAÑO_PLEXOBRAQUIAL	SI	Recuento	1	0	1
		% dentro de GRUPO_ALEATORIZADO	,2%	,0%	,1%
	NO	Recuento	439	434	873
		% dentro de GRUPO_ALEATORIZADO	99,8%	100,0%	99,9%
Total		Recuento	440	434	874
		% dentro de GRUPO_ALEATORIZADO	100,0%	100,0%	100,0%

Pruebas de chi-cuadrado

	Valor	gl	Sig. asintótica (bilateral)	Sig. exacta (bilateral)	Sig. exacta (unilateral)
Chi-cuadrado de Pearson	,987[a]	1	,320		
Corrección por continuidad[b]	,000	1	1,000		
Razón de verosimilitudes	1,374	1	,241		
Estadístico exacto de Fisher				1,000	,503
Asociación lineal por lineal	,986	1	,321		
N de casos válidos	874				

a. 2 casillas (50,0%) tienen una frecuencia esperada inferior a 5. La frecuencia mínima esperada es ,50.

b. Calculado sólo para una tabla de 2x2.

PREMATURO <37 VS >=37 * GRUPO_ALEATORIZADO

Tabla de contingencia

			GRUPO_ALEATORIZADO		
			CONTROL	INTERVENCION ACEITE OLIVA	Total
PREMATURO <37 VS >=37	1,00	Recuento	17	5	22
		% dentro de GRUPO_ALEATORIZADO	3,9%	1,2%	2,5%
	2,00	Recuento	423	429	852
		% dentro de GRUPO_ALEATORIZADO	96,1%	98,8%	97,5%
Total		Recuento	440	434	874
		% dentro de GRUPO_ALEATORIZADO	100,0%	100,0%	100,0%

Pruebas de chi-cuadrado

	Valor	gl	Sig. asintótica (bilateral)	Sig. exacta (bilateral)	Sig. exacta (unilateral)
Chi-cuadrado de Pearson	6,547[a]	1	,011		
Corrección por continuidad[b]	5,488	1	,019		
Razón de verosimilitudes	6,917	1	,009		
Estadístico exacto de Fisher				,016	,009
Asociación lineal por lineal	6,539	1	,011		
N de casos válidos	874				

a. 0 casillas (,0%) tienen una frecuencia esperada inferior a 5. La frecuencia mínima esperada es 10,92.

b. Calculado sólo para una tabla de 2x2.

LGA> 90 PERCENTILE * GRUPO_ALEATORIZADO

Tabla de contingencia

			GRUPO_ALEATORIZADO		
			CONTROL	INTERVENCION ACEITE OLIVA	Total
LGA> 90 PERCENTILE	1,00	Recuento	18	4	22
		% dentro de GRUPO_ALEATORIZADO	4,1%	,9%	2,5%

	2,00	Recuento	422	430	852
		% dentro de GRUPO_ALEATORIZADO	95,9%	99,1%	97,5%
Total		Recuento	440	434	874
		% dentro de GRUPO_ALEATORIZADO	100,0%	100,0%	100,0%

Pruebas de chi-cuadrado

	Valor	gl	Sig. asintótica (bilateral)	Sig. exacta (bilateral)	Sig. exacta (unilateral)
Chi-cuadrado de Pearson	8,943[a]	1	,003		
Corrección por continuidad[b]	7,699	1	,006		
Razón de verosimilitudes	9,670	1	,002		
Estadístico exacto de Fisher				,004	,002
Asociación lineal por lineal	8,933	1	,003		
N de casos válidos	874				

a. 0 casillas (,0%) tienen una frecuencia esperada inferior a 5. La frecuencia mínima esperada es 10,92.

b. Calculado sólo para una tabla de 2x2.

SGA < 10 PERCENTILE * GRUPO_ALEATORIZADO

Tabla de contingencia

			GRUPO_ALEATORIZADO		
			CONTROL	INTERVENCION ACEITE OLIVA	Total
SGA < 10 PERCENTILE	1,00	Recuento	25	5	30
		% dentro de GRUPO_ALEATORIZADO	5,7%	1,2%	3,4%
	2,00	Recuento	415	429	844
		% dentro de GRUPO_ALEATORIZADO	94,3%	98,8%	96,6%
Total		Recuento	440	434	874
		% dentro de GRUPO_ALEATORIZADO	100,0%	100,0%	100,0%

Pruebas de chi-cuadrado

	Valor	gl	Sig. asintótica (bilateral)	Sig. exacta (bilateral)	Sig. exacta (unilateral)
Chi-cuadrado de Pearson	13,525[a]	1	,000		
Corrección por continuidad[b]	12,193	1	,000		
Razón de verosimilitudes	14,746	1	,000		
Estadístico exacto de Fisher				,000	,000
Asociación lineal por lineal	13,510	1	,000		
N de casos válidos	874				

a. 0 casillas (,0%) tienen una frecuencia esperada inferior a 5. La frecuencia mínima esperada es 14,90.

b. Calculado sólo para una tabla de 2x2.

HIPOGLUCEMIA * GRUPO_ALEATORIZADO

Tabla de contingencia

			GRUPO_ALEATORIZADO		
			CONTROL	INTERVENCION ACEITE OLIVA	Total
HIPOGLUCEMIA	SI	Recuento	9	3	12
		% dentro de GRUPO_ALEATORIZADO	2,0%	,7%	1,4%
	NO	Recuento	431	431	862
		% dentro de GRUPO_ALEATORIZADO	98,0%	99,3%	98,6%
Total		Recuento	440	434	874
		% dentro de GRUPO_ALEATORIZADO	100,0%	100,0%	100,0%

Pruebas de chi-cuadrado

	Valor	gl	Sig. asintótica (bilateral)	Sig. exacta (bilateral)	Sig. exacta (unilateral)
Chi-cuadrado de Pearson	2,959[a]	1	,085		

Corrección por continuidad[b]	2,043	1	,153		
Razón de verosimilitudes	3,098	1	,078		
Estadístico exacto de Fisher				,143	,075
Asociación lineal por lineal	2,956	1	,086		
N de casos válidos	874				

a. 0 casillas (,0%) tienen una frecuencia esperada inferior a 5. La frecuencia mínima esperada es 5,96.

b. Calculado sólo para una tabla de 2x2.

DISTRES_RESPIRATORIO * GRUPO_ALEATORIZADO

Tabla de contingencia

			GRUPO_ALEATORIZADO		Total
			CONTROL	INTERVENCION ACEITE OLIVA	
DISTRES_RESPIRATORIO	SI	Recuento	4	3	7
		% dentro de GRUPO_ALEATORIZADO	,9%	,7%	,8%
	NO	Recuento	436	431	867
		% dentro de GRUPO_ALEATORIZADO	99,1%	99,3%	99,2%
Total		Recuento	440	434	874
		% dentro de GRUPO_ALEATORIZADO	100,0%	100,0%	100,0%

Pruebas de chi-cuadrado

	Valor	gl	Sig. asintótica (bilateral)	Sig. exacta (bilateral)	Sig. exacta (unilateral)
Chi-cuadrado de Pearson	,131[a]	1	,718		
Corrección por continuidad[b]	,000	1	1,000		
Razón de verosimilitudes	,131	1	,717		
Estadístico exacto de Fisher				1,000	,508
Asociación lineal por lineal	,130	1	,718		
N de casos válidos	874				

Pruebas de chi-cuadrado

	Valor	gl	Sig. asintótica (bilateral)	Sig. exacta (bilateral)	Sig. exacta (unilateral)
Chi-cuadrado de Pearson	,131[a]	1	,718		
Corrección por continuidad[b]	,000	1	1,000		
Razón de verosimilitudes	,131	1	,717		
Estadístico exacto de Fisher				1,000	,508
Asociación lineal por lineal	,130	1	,718		
N de casos válidos	874				

a. 2 casillas (50,0%) tienen una frecuencia esperada inferior a 5. La frecuencia mínima esperada es 3,48.

b. Calculado sólo para una tabla de 2x2.

HPERBILIRRUNEMIA_YES_VS_NO * GRUPO_ALEATORIZADO

Tabla de contingencia

			GRUPO_ALEATORIZADO		
			CONTROL	INTERVENCION ACEITE OLIVA	Total
HPERBILIRRUNEMIA_YES_VS_NO	1,00	Recuento	31	22	53
		% dentro de GRUPO_ALEATORIZADO	7,0%	5,1%	6,1%
	2,00	Recuento	409	412	821
		% dentro de GRUPO_ALEATORIZADO	93,0%	94,9%	93,9%
Total		Recuento	440	434	874
		% dentro de GRUPO_ALEATORIZADO	100,0%	100,0%	100,0%

Pruebas de chi-cuadrado

	Valor	gl	Sig. asintótica (bilateral)	Sig. exacta (bilateral)	Sig. exacta (unilateral)
Chi-cuadrado de Pearson	1,498[a]	1	,221		
Corrección por continuidad[b]	1,171	1	,279		
Razón de verosimilitudes	1,506	1	,220		

Estadístico exacto de Fisher				,257	,140
Asociación lineal por lineal	1,496	1	,221		
N de casos válidos	874				

a. 0 casillas (,0%) tienen una frecuencia esperada inferior a 5. La frecuencia mínima esperada es 26,32.

b. Calculado sólo para una tabla de 2x2.

NICU_OBSERVATION_STANCE * GRUPO_ALEATORIZADO

Tabla de contingencia

			GRUPO_ALEATORIZADO		
			CONTROL	INTERVENCION ACEITE OLIVA	Total
NICU_OBSERVATION_STANCE	1,00	Recuento	14	8	22
		% dentro de GRUPO_ALEATORIZADO	3,2%	1,8%	2,5%
	2,00	Recuento	426	426	852
		% dentro de GRUPO_ALEATORIZADO	96,8%	98,2%	97,5%
Total		Recuento	440	434	874
		% dentro de GRUPO_ALEATORIZADO	100,0%	100,0%	100,0%

Pruebas de chi-cuadrado

	Valor	gl	Sig. asintótica (bilateral)	Sig. exacta (bilateral)	Sig. exacta (unilateral)
Chi-cuadrado de Pearson	1,595[a]	1	,207		
Corrección por continuidad[b]	1,096	1	,295		
Razón de verosimilitudes	1,616	1	,204		
Estadístico exacto de Fisher				,280	,147
Asociación lineal por lineal	1,593	1	,207		
N de casos válidos	874				

a. 0 casillas (,0%) tienen una frecuencia esperada inferior a 5. La frecuencia mínima esperada es 10,92.

b. Calculado sólo para una tabla de 2x2.

Zou G. A Modified Poisson Regression Approach to Prospective Studies with Binary Data. Am J Epidemiol 2004; 159(7):702-6.

GESTACIONAL DIABETES MELLITUS						
	Zhang and Yu: RR(95%CI)	p	Poisson regression with robust error variance: RR(95%CI)	p	Cox regression with robust variance: RR (95% CI)	p
Crude	0.73 (0.55-0.95)	0.020	0.73 (0.56-0.95)	0.020	0.73 (0.56-0.95)	0.020
Model 1	0.72 (0.54-0.95)	0.018	0.73 (0.55-0.95)	0.019	0.73 (0.55-0.95)	0.019
Model 2	0.74 (0.55-0.98)	0.037	0.75 (0.58-0.99)	0.041	0.75 (0.57-0.99)	0.041
Modelo 3	0.73 (0.55-0.96)	0.021	0.73 (0.56-0.97)	0.022	0.73 (0.56-0.96)	0.022
Model 1 +2	0.73 (0.54-0.97)	0.031	0.74 (0.56-0.97)	0.033	0.74 (0.57-0.98)	0.033
Modelo1 + 2 + 3	0.74 (0.55-0.98)	0.037	0.75 (0.57-0.98)	0.039	0.75 (0.57-0.98)	0.039

Model 1: adjusted for age (continuous), ethnicity and parity.
Model 2: adjusted for BMI (continuous).
Model 3: adjusted for gestacional, personal and family history, smoker (categorical: never, current, former smoker).

PREMATUTITY						
	Zhang and Yu: RR(95%CI)	p	Poisson regression with robust error variance: RR(95%CI)	p	Cox regression with robust variance: RR (95% CI)	p
Crude	0.27 (0.10-0.75)	0.011	0.28 (0.10-0.75)	0.011	0.28 (0.11-0.75)	0.011
Model 1	0.29 (0.11-0.78)	0.014	0.30 (0.11-0.79)	0.016	0.30 (0.11-0.79)	0.016
Model 2	0.34 (0.12-0.94)	0.038	0.34 (0.12-0.96)	0.041	0.34 (0.12-0.96)	0.041
Modelo 3	0.29 (0.11-0.77)	0.013	0.29 (0.11-0.77)	0.013	0.29 (0.11-0.77)	0.013

Model 1: adjusted for age (continuous), ethnicity and parity.
Model 2: adjusted for BMI (continuous).
Model 3: adjusted for gestacional, personal and family history, smoker (categorical: never, current, former smoker).

LARGE FOR GESTACIONAL AGE						
	Zhang and Yu: RR(95%CI)	p	Poisson regression with robust error variance: RR(95%CI)	p	Cox regression with robust variance: RR (95% CI)	p
Crude	0.20 (0.07-0.59)	0.003	0.20 (0.07-0.59)	0.003	0.20 (0.07-0.59)	0.003
Model 1	0.21 (0.07-0.60)	0.004	0.21 (0.07-0.60)	0.004	0.21 (0.07-0.30)	0.004
Model 2	0.24 (0.08-0.70)	0.009	0.24 (0.08-0.71)	0.011	0.24 (0.08-0.72)	0.011
Modelo 3	0.20 (0.07-0.58)	0.002	0.19 (0.07-0.57)	0.003	0.20 (0.07-0.57)	0.003

Model 1: adjusted for age (continuous), ethnicity and parity.
Model 2: adjusted for BMI (continuous).
Model 3: adjusted for gestacional, personal and family history, smoker (categorical: never, current, former smoker).

SMALL FOR GESTATIONAL AGE						
	Zhang and Yu: RR(95%CI)	p	Poisson regression with robust error variance: RR(95%CI)	p	Cox regression with robust variance: RR (95% CI)	p
Crude	0.20 (0.08-0.52)	0.001	0.20 (0.08-0.52)	0.001	0.20 (0.08-0.52)	0.001
Model 1	0.20 (0.08-0.52)	0.001	0.20 (0.08-0.53)	0.001	0.20 (0.08-0.53)	0.001

Model 2	0.22 (0.08-0.58)	0.002	0.22 (0.09-0.57)	0.002	0.22 (0.09-0.57)	0.002
Modelo 3	0.21 (0.08-0.54)	0.001	0.21 (0.08-0.54)	0.001	0.21 (0.08-0.54)	0.001

Model 1: adjusted for age (continuous), ethnicity and parity.
Model 2: adjusted for BMI (continuous).
Model 3: adjusted for gestacional, personal and family history, smoker (categorical: never, current, former smoker).

PERIANAL TRAUMA						
	Zhang and Yu: RR(95%CI)	p	Poisson regression with robust error variance: RR(95%CI)	p	Cox regression with robust variance: RR (95% CI)	p
Crude	0.20 (0.08-0.53)	<0.001	0.21 (0.12-0.36)	<0.001	0.21 (0.12-0.36)	<0.001
Model 1	0.20 (0.08-0.52)	<0.001	0.20 (0.11-0.35)	<0.001	0.20 (0.11-0.35)	<0.001
Model 2	0.22 (0.08-0.59)	<0.001	0.21 (0.12-0.37)	<0.001	0.21 (0.12-0.37)	<0.001
Modelo 3	0.21 (0.08-0.54)	<0.001	0.21 (0.12-0.36)	<0.001	0.21 (0.12-0.36)	<0.001

Model 1: adjusted for age (continuous), ethnicity and parity.
Model 2: adjusted for BMI (continuous)
Model 3: adjusted for gestacional, personal and family history, smoker (categorical: never, current, former smoker).

EMERGENCY CESAREAN						
	Zhang and Yu: RR(95%CI)	p	Poisson regression with robust error variance: RR(95%CI)	p	Cox regression with robust variance: RR (95% CI)	p
Crude	0.29 (0.14-0.61)	0.001	0.29 (0.14-0.61)	0.001	0.29 (0.14-0.61)	0.001
Model 1	0.28 (0.13-0.58)	0.001	0.28 (0.14-0.59)	0.001	0.28 (0.14-0.58)	0.001
Model 2	0.32 (0.15-0.67)	0.002	0.32 (0.16-0.67)	0.003	0.32 (0.16-0.67)	0.003
Modelo 3	0.30 (0.14-0.62)	0.001	0.30 (0.14-0.63)	0.001	0.30 (0.1-0.63)	0.001

Model 1: adjusted for age (continuous), ethnicity and parity.
Model 2: adjusted for BMI (continuous).
Model 3: adjusted for gestacional, personal and family history, smoker (categorical: never, current, former smoker).

URINARY TRACT INFECTION						
	Zhang and Yu: RR(95%CI)	p	Poisson regression with robust error variance: RR(95%CI)	p	Cox regression with robust variance: RR (95% CI)	p
Crude	0.40 (0.25-0.63)	<0.001	0.40 (0.25-0.63)	<0.001	0.40 (0.25-0.63)	<0.001
Model 1	0.40 (0.25-0.64)	<0.001	0.41 (0.26-0.64)	<0.001	0.41 (0.26-0.64)	<0.001
Model 2	0.41 (0.25-0.65)	<0.001	0.41 (0.26-0.65)	<0.001	0.41 (0.26-0.65)	<0.001
Modelo 3	0.41 (0.25-0.64)	<0.001	0.41 (0.26-0.64)	<0.001	0.41 (0.26-0.64)	<0.001

Model 1: adjusted for age (continuous), ethnicity and parity.
Model 2: adjusted for BMI (continuous).
Model 3: adjusted for gestacional, personal and family history, smoker (categorical: never, current, former smoker).

INSULIN-TREATED GDM						
	Zhang and Yu: RR(95%CI)	p	Poisson regression with robust error variance:	p	Cox regression with robust variance: RR (95% CI)	p

			RR(95%CI)			
Crude	0.43 (0.24-0.78)	0.005	0.43 (0.24-0.78)	0.006	0.43 (0.24-0.78)	0.006
Model 1	0.45 (0.24-0.80)	0.007	0.44 (0.25-0.81)	0.008	0.44 (0.25-0.81)	0.008
Model 2	0.50 (0.27-0.91)	0.024	0.51 (0.28-0.93)	0.028	0.51 (0.28-0.93)	0.028
Modelo 3	0.41 (0.24-0.78)	0.005	0.43 (0.24-0.78)	0.005	0.43 (0.24-0.78)	0.005

Model 1: adjusted for age (continuous), ethnicity and parity.
Model 2: adjusted for BMI (continuous).
Model 3: adjusted for gestacional, personal and family history, smoker (categorical: never, current, former smoker).

The methods used offer estimations of the effect size (RR) and intervals confidence precisions very close. The conclusions from the point of view of statistical significance are the same.
