# Supplementary material for: A Mediterranean diet with additional extra virgin olive oil and pistachios reduces the incidence of gestational diabetes mellitus (GDM): A randomized controlled trial: The St. Carlos GDM prevention study
Source: PLoS One. 2017 Oct 19;12(10):e0185873. doi: 10.1371/journal.pone.0185873 (PMC5648128; doi:10.1371/journal.pone.0185873)
Supplement: S2 File — English version. (PDF) [file pone.0185873.s002.pdf]

ORIGINAL PROTOCOL :

Reduction in the incidence of gestational diabetes mellitus (GDM) with MedDiet and its impact on genetic and epigenetic pattern expression in pregnant women and their offspring.

Summary of the project……………………………………. 2

Background………………………………………………… 3

Study hypothesis and Objectives…………………………...5

Summary Intervention…………………………………….. 6

Sample size calculation…………………………………….. 6

Selection of Patients………………………………………. . 7

Flowchart……………………………………………………8

Intervention and Follow-up……………………………….8

GDM treatment……………………………………………10

Team………………………………………………………12

Lifestyle questionnaire…………………………………..13

Statistical analysis………………………………………..14

Ethic, deontological and regulatory considerations……15

ADENDUM, November 30, 2014………………………16

Final statistical analysis…………………………..…….17

ADENDUM April 01, 2017..18

ADENDUM, May 4, 2017...19

SUMMARY OF THE MOST IMPORTANT GOALS OF THE PROJECT PROPOSAL: Gestational diabetes mellitus (GDM) is a growing health problem associated with adverse pregnancy and birth outcomes. It is also considered an established risk factor for the development of type 2 diabetes and cardiovascular disease over longer term for both women and the offspring. Its incidence is increasing, not only as a result of the application of more stringent criteria of diagnosing. In the last years, there has been a dramatic increase in the prevalence of prepregnancy obesity, a main risk factor for maternal and neonatal morbidity and mortality, as a direct consequence of the global obesity epidemic. Recent data suggest that the prevalence of obesity in reproductive-age women is around 10% in Spanish women. The origin of this epidemic is an unhealthy lifestyle, with high-energy and high-fat containing diets, physical inactivity and smoking. Of interest, there is now substantial evidence for the importance of the intrauterine environment on the health and longevity of the offspring, although the mechanisms underlying remain poorly understood. The Mediterranean Diet (MedDiet) has been associated with greater longevity and quality of life, and with potential advantages in the prevention of cardiovascular diseases. Thus, in this project we will attempt to study the effect of a lifestyle intervention based on a MedDiet and physical activity/exercise performance, beginning after the 1st gestational visit [8-12 gestational weeks (GWs)] and throughout the pregnancy, in the incidence of GDM rate. In addition, we will investigate its impact on genetic and epigenetic (DNA methylation and microRNAs expression) pattern expression, and gut microbiome composition both in women and in the offspring. Since GDM evolves through loops of deregulated inflammation, genetic/epigenetic modifications, gut microbiota and diet, we believe that the synergic analysis of such parameters could provide new biological insights and effective biomarkers that could have applications in prevention, molecular diagnosis and prognosis of GDM. The idea of preventing, or even managing, diseases such as diabetes or obesity with diet is very attractive but deserves further studies.

BACKGROUND

Several modifiable risk factors related to the onset of type 2 Diabetes in women have been identified (1), and lifestyle changes, particularly the Mediterranean Diet (MedDiet), have proved to be effective in preventing its occurrence (2-3).

Gestational diabetes mellitus (GDM) is a growing health problem because of its increasing prevalence, its association with adverse pregnancy and birth outcomes (4) and because is an established risk factor for the development of type 2 diabetes and cardiovascular disease over longer term (5). The use of new criteria for diagnosing GDM, recommended after the HAPO study (6), has been associated with an increase in prevalence, i.e. from 8% (NDDG criteria) and 12.5% (Carpenter Coustan criteria) to 35% in a revaluation of our population of women (7). Despite the diagnosis of GDM with more stringent criteria, HAPO study shows a continuous association of maternal glucose levels below those previously used to GDM diagnosis with increased birth weight and adverse pregnancy outcomes. Regarding this dramatic increase in GDM diagnosis observed with the proposed IADPSG criteria, it is important to remember that previous criteria required two abnormal oral glucose tolerance tests. However, it has been demonstrated that women who had one abnormal test value and who were considered previously as "normal" had many of the outcomes that were similar to those who were as GDM.

Recently, based in a large population of more than 2000 women, our group has developed a risk appraisal model for GDM by identifying modifiable factors that can help to predict the risk of GDM (7). After logistic regression model, biscuits and pastries <4 times/week, red and processed meats intake < 6 serving/week, juices < 4 servings/ week, light walking >30 minutes/day and 30 minutes/day of physical activity of moderate intensity at least 2 days/week was associated with less GDM risk compared to the opposite. This data may represent a promising approach for the prevention of GDM and subsequent complications. In Spain, the Community of Madrid is one of the regions with higher foreigner rates, 18.8% of the total population (January 2011), with 49.7% of women. About 50% of pregnant women attended in our hospital during 2012 were immigrants. Recently, we have reported that this population tends to acquire the same risk factors as the Spanish population associated with lifestyle (8). Prepregnancy

maternal obesity is a major risk factor for maternal and neonatal morbidity and mortality, including GDM (9). Adipokines and inflammatory mediators are considered the main factors causing chronic subclinical inflammation that leads to insulin resistance and abnormality in glucose metabolism (9,10).

Additionally, there is increasing evidence that exposure to an adverse fetal and/or early postnatal environment may increase susceptibility to a number of chronic diseases later in the life of the offspring. Many studies have shown that offspring of diabetic mothers have a statistically higher incidence of impaired glucose tolerance, a well-known prediabetic state (11). In the same way, the incidence of diabetes is higher in all age groups of offspring who are exposed to hyperglycemia in uterus compared with offspring of nondiabetic mothers, even though some of them would later still develop diabetes mellitus (11). Therefore, diabetes in the offspring is mainly the consequence of exposure to a diabetic intrauterine environment, in conjunction with genetic susceptibility. Thus, there is great interest in identifying the exact mechanisms by which maternal hyperglycemia may lead to diseases in the offspring later in life in aims to develop strategies to prevent this destructive cycle of metabolic dysfunction through generations.

1. Hu FB et al. Diet life style and the risk of type 2 diabetes mellitus in women. N Engl J Med 2001;345:790-7

2. Salas-Salvadó J et al. Reduction in the incidence of type 2 diabetes with the Mediterranean diet: results of the PREDIMED-Reus nutrition intervention randomized trial. Diabetes Care 2011;34:14-9

3. The InterAct Consortium et al. Mediterranean Diet and Type2 Diabetes Risk in the European Prospective Investigation into Cancer and Nutrition (EPIC) Study: the InterAct project. Diabetes Care 2011;34:1913-8

4. Casey BM et al. Pregnancy outcomes in women with gestational diabetes compared with the general obstetric population. Obstet Gynecol 1997;90: 869-73

5. Kim C et al. Gestational diabetes and the incidence of type 2 diabetes: A systematic review. Diabetes Care 2002;25:1862-8

6. The HAPO Study Group. Hyperglycemia and Adverse Pregnancy Outcomes N Engl J Med 2008;358:1991-2002

7. Ramos-Levi A et al. Risk factors for gestational diabetes mellitus in a large population of women living in Spain: Implications for preventative strategies. Int J Endocrinol 2012:312529

8. Pérez-Ferre N et al. Effect of lifestyle on the risk of gestational diabetes and obstetric outcomes in immigrant Hispanic women living in Spain. J Diabetes 2012;4:432-8
9. Guelinckx I et al. Maternal obesity: pregnancy complications, gestational weight gain and nutrition. Obes Rev 2008;9:140-50
10. Vrachnis N et al. Role of adipokines and other inflammatory mediators in gestational diabetes mellitus and previous gestational diabetes mellitus. Int J Endocrinol 2012:549748
11. Metzger BE. Long-term outcomes in mothers diagnosed with gestational diabetes mellitus and their offspring. Clin Obstet Gynecol. 2007;50(4):972-9

STUDY HYPOTHESIS: Traditionally, it has been widely accepted that genetic and adult lifestyle factors determine our risk of developing some diseases such as obesity, diabetes and cardiovascular disease in later life. However, there is now substantial evidence of the importance of the intrauterine environment in determining our susceptibility to such diseases in later life, although the mechanisms underlying remain poorly understood. In addition, one cannot consider genetic determinants of human metabolism without considering the influence of dietary components metabolized by gut microbiota. Therefore, in this project we have hypothesized that a lifestyle intervention based on the MedDiet and physical activity/exercise performance, beginning after 1st gestational visit [8-12 gestational weeks (GWs)] and throughout the pregnancy, will reduce the incidence of gestational diabetes mellitus (GDM) in women with normal fasting plasma glucose (FPG). Moreover, the mechanism by which MedDiet/physical activity could alter long term disease risk in both women and their offspring may involve epigenetic processes or alterations in gut microbiome composition.

OBJECTIVES:

Primary outcome:

- To define the prevention of GDM (evaluated at 24-28 GWs with HAPO criteria) after the lifestyle intervention based on MedDiet and physical activity/exercise, as compared to standard treatment, in women with normal FPG (<92mg/dL) at the 1st gestational visit (8-12 GWs).

Secondary outcome:

- To define functional genetic risk of developing GDM focusing in obese and non obese pregnant women.
- To investigate the effects of MedDiet and physical exercise/activity scheduled in inflammatory biomarkers in pregnant women.
- To investigate the effects of MedDiet and physical exercise/activity scheduled in the epigenetic mechanisms (DNA methylation and miRNA expression) in pregnant women and offspring; if so, it would represent a novel epigenetic mechanism for regulation of gene expression in the offspring.
- To investigate whether the MedDiet and physical exercise/activity scheduled alters the composition of the gut microbial flora both in women and offspring.
- To evaluate the impact of all these parameters on the long-term health of newborns in the first year of life.
- To estimate changes in HbA1c, insulin, HOMA, lipid profile, body weight, blood pressure, adherence to changes in lifestyle estimated by Med Score and urinary hydroxytyrosol levels and plasma γ-tocopherol levels to confirm compliance in the group receiving MedDiet [extra virgin olive oil (EVOO) and pistachios].
- The parameters to be assessed will be: gestation duration and fetal development; delivery characteristics such as caesarean delivery and instrumental vaginal birth, placental weight; and newborn data such as newborn weight, Apgar test values and cord blood pH.

Summary Intervention

Strategies for preventing GDM have until now been focused on diet and exercise interventions, which have had mixed results. This could be possibly because many of these trials have studied small sample sizes. A recent randomized controlled trial of MedDiet intervention (PREDIMED Study) has reported a decline in fasting plasma glucose [-7 (IQR:-13; -1.3) mg/dl] after 3 months in a Mediterranean population at high cardiovascular risk. These data could represent a potential reduction of GDM rate of more than 30%, although this aspect deserves to be demonstrated. The project proposed is a large, prospective, randomized, clinic-based, interventional study with two parallel groups that will provide to answer this question.

SAMPLE SIZE CALCULATION

For sample size calculation, a primary end-point for conversion to GDM has been used, assuming an expected conversion rate of 35% between 24-28 GWs and a reduction of at least 30% in the conversion to GDM after intervention. This intervention has shown a decrease of FPG -7 (-13; -1.3) mg/dl after 3 months in the PREDIMED Study (1), representing a potential reduction of GDM rate higher than 30%. A sample size of 315 women analyzed per group to provide statistical power of 80% to detect a 30% difference between groups in the primary outcomes at 5% significance. This number has been increased to **500 per group**, to allow for a predicted drop-out of around 20% (in accordance with the expected response rate, loss to follow up, lack of compliance, and any other predicted reasons for loss of subjects). Therefore, **1000 women will be randomized** in the study and we expect to assess at least 700.

Selection of Patients

The Institutional Review Board and the Clinical Ethic Committee of the Hospital Clínico San Carlos have approved this protocol before initiation of the study (approval reference: CP.CI.13/296-E). We will include pregnant women who fulfill the inclusion criteria and none of the exclusion criteria. Subjects must give signed and dated informed consent (attached in a separate file) prior to any trial-related activities. Structure of the trial is displayed in ***Figure 1***.

Inclusion criteria: women >18 year old, with normal fasting glucose values (< 92 mg/dl) in the 1st gestational assessment (8-12 GWs) and sign the informed consent.

Exclusion criteria: women with fasting glucose levels ≥92 mg/dl in the 1st gestational assessment (8-12 GWs), multiple pregnancy, nut allergy, or any other reason, medical condition, ongoing medication or significant disability that would prevent the participant complying with trial consent, treatment and follow-up procedures or potentially jeopardize her medical care.

Figure 1 - Flowchart of the trial.

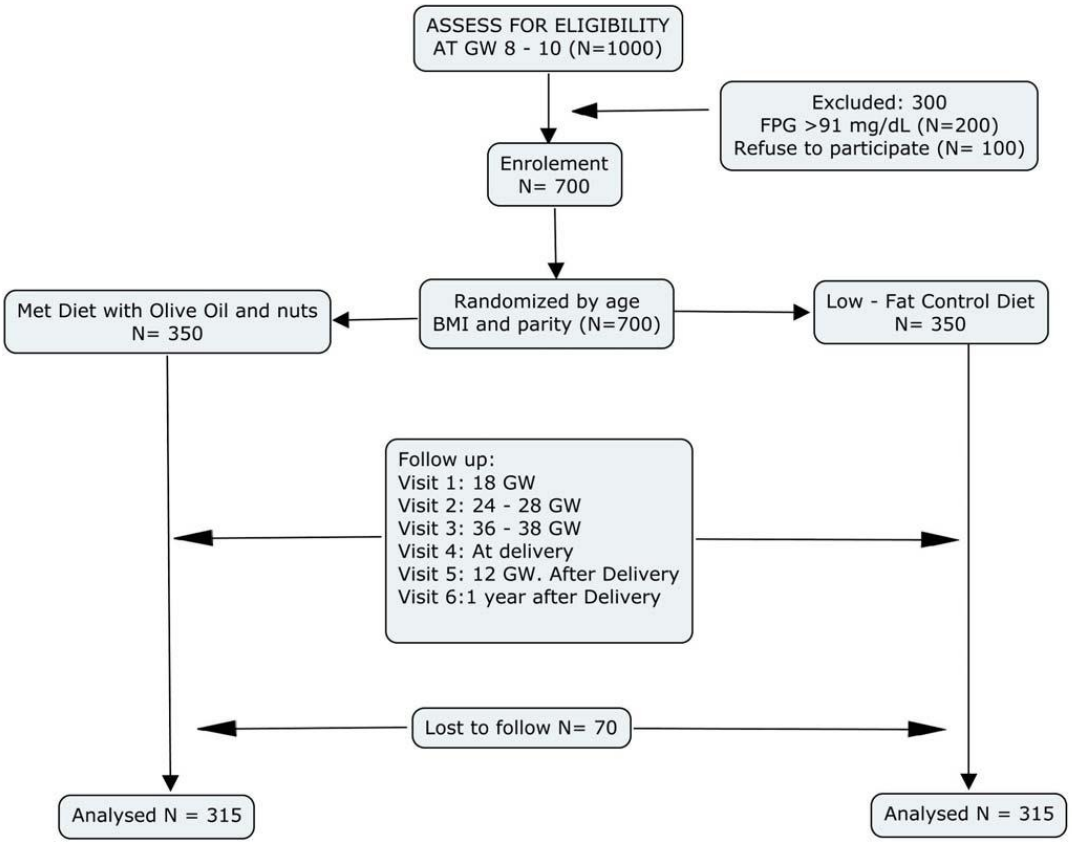

Intervention

Eligible women will randomly be assigned to the:

- Control group (CG). Women will be assigned to a standard treatment based on recommendations provided in clinical practice. The main recommendation is to reduce all types of fat from both animal and vegetable sources including nuts and EVOO, and a training program is also provided. This prudent diet recommendation represents a contribution of total fat less than 30% of total energy intake (<40 mL EVOO and not nuts consumption), and carbohydrate intake more than 50%. Women will be followed up by the Obstetric Department.

- Intervention group (IG). Women with free allotments of EVOO (1 liter/week) and pistachios (150 g/week), and the training program. Dieticians will give personalized

dietary advice to participants with instructions directed to use of EVOO for cooking and dressing, increased consumption of fruit, vegetables, legumes, and fish and avoidance of red or processed meat, butter, cream, fast food, sweets, pastries, and sugar-sweetened beverages. This intervention diet is comprised of an intake of approximately 35-40% of total fat (predominantly unsaturated fatty acids) and 40-45% of low glycemic index carbohydrates, maintaining a protein intake of 20%, similar to the control diet. Nutrition intervention is aimed to achieve a lifestyle score >10 or Nutrition Score >8 based on Diabetes Nutrition and Complications Trial (DNCT) previously reported. The questionnaire is displayed in **Table 1**. Women will be followed up by the Endocrinology Department.

The main investigator will construct a Master Randomization List using a permuted block design. Women will be stratified by age, BMI and parity and allocation will be assigned in a ratio of 1:1 in random blocks of 4 and 6.

Follow-up

Women will be enrolled at 8-12 GWs (visit 0) and will follow-up at GWs 12-14 (visit 1), 24-28 (visit 2), 34-36 (visit 3) and delivery (visit 4), as well as at 12 weeks (visit 5) and 1 year post delivery (visit 6).

Baseline assessments (Visit 0): Women will be evaluated after the signature of the informed consent form. A complete medical history and physical exam will be performed. Subsequent assessments (Visits 1-6): Women will be followed up by means of clinic visits by the study staff. Six clinic visits will be performed, including: assessment of endpoints/outcome measures, assessments of safety including general (e.g. physical examination), specific safety assessments (e.g. blood chemistries including general biochemistry, hematology and urine analyses) and adverse event collection, assessment of compliance with the study and recording of concomitant medications. Blood and stool samples will be taken at this time. At visit 5 and 6, cord blood and placenta will be taken.

All biological samples will be extracted in one point and will be supervised by a scientist staff that will ensure fit for-purpose operations (high-throughput, high-quality). Design and testing of the sample-handling protocol will consider key factors that could affect the stability of biological samples, including anti-coagulants, stabilizing agents, temperature, and elapsed time from collection to initial processing and endogenous

degrading properties (enzymes). Sample fractions will be stored in two separated ultra-low temperature freezers to ensure long-term stability and integrity.

GDM TREATMENT

Women from both groups diagnosed with GDM will be referred to the Diabetes and Pregnancy Unit and treated according to local guidelines previously reported (The St. Carlos Gestational Diabetes Study. Diabetes Care 2014;37:2442-50). ***Figure 2*** shows a flowchart of GDM treatment protocol.

Lifestyle intervention: Specific diet was not provided to the women, just some recommendations on Lifestyle. Women will be advised to avoid or limit the intake of refined carbohydrates, sugary drinks including fresh fruit juices, red meat (particularly processed meat), bakery and pastries. At least 3 servings of skimmed dairy products per day, 2 servings of vegetables per day, and 2 servings of fresh fruit (excluding juices) per day will be recommended. Nuts instead of processed meat snack; oily fish and EVOO daily; and whole grain cereals and legumes instead of white cereals and potatoes will also recommended.

A physically active lifestyle including walking at least 15 minutes, and climbing stairs at least 4 floors, 4 times a day > 5 days per week, will be recommended.

A semi quantitative questionnaire was used to collect lifestyle adherence to these recommendations and displayed. The goal will be to achieve score >8

Figure 2 - GDM treatment.

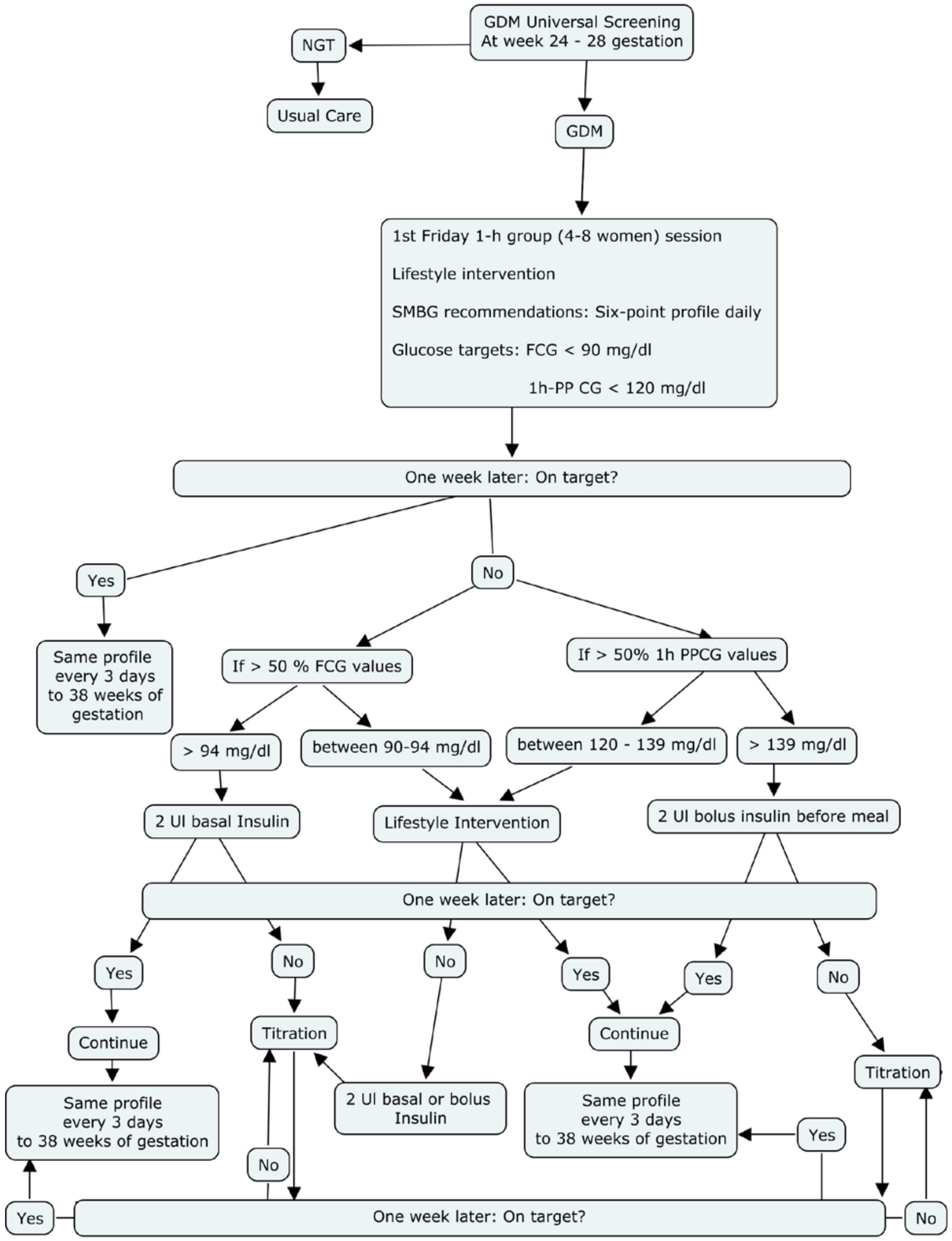

TEAM.

The Department of Endocrinology and Metabolic Diseases of the Hospital Clínico San Carlos is a leading research group in the field of prevention and epidemiology of diabetes in Spain. Its personnel have been involved in various landmark projects on public health aspects of diabetes, and cardiovascular diseases, in Spain and in many other countries. In the last years, the group has developed an interest in GDM, establishing a close collaboration with the Department of Gynaecology and Obstetrics of the Hospital Clínico San Carlos.

In this project, each gynaecologist/obstetric investigator will be responsible for recruiting patients, asking for and obtaining written informed consent, evaluating inclusion and exclusion criteria and notifying the patient inclusion to the coordinator. They will also be responsible of random group assignment, program patient visits according to the study protocol and data collection. For this task, age, sex, education level (no studies, elementary, secondary, technical school or university graduate), marital status (single, divorced, married or living together as a couple or widowed) smoking habits (current vs. non-smoker), leisure-time sports (days per week and hours per day), family history of diabetes or metabolic syndrome, personal obstetric history and history of hypertension, dyslipidemia or coronary heart disease will be recorded. Weight, height and waist and hip circumferences will be directly measured by the research physician using standardized methods. Since women who developed GDM will need intensive blood sugar control to prevent maternal and fetal complications and treatment with dietary or medication therapy based on their individual needs, endocrinologists will follow them. They will be simultaneously followed by their obstetricians for appropriate monitoring of the pregnancy. A dietician will carry out lifestyle interventions for all women. They will be developed during a 1-h session (Visit 1) for each woman individually and will be reinforced at each follow-up visit. We have developed a questionnaire to evaluate adherence to recommended lifestyle changes (***Table 1***). A score of +1, 0 or -1 has been assigned to each of the 18 items on the questionnaire, with a value of +1 indicating that the beneficial recommendation is regularly performed, -1 indicating that the beneficial recommendation has not been adopted or that patients are persisting with an unhealthy habit, and 0 indicating

intermediate consumptions or exercise frequency between healthy and unhealthy adults. Different composite variables from the questionnaire will assess overall Lifestyle, including a Physical Activity score (items 1 -3), a Nutrition score (items 4 -15), a Low Glycaemic Index score (items 4, 5, 9, and 10), an Unsaturated Fat score (items 6 -8).

Table 1: Lifestyle questionnaire.

Physical Activity	***+1***	***0***	***1***	**+1, 0 or -1**
Walking daily	+ 1 h	30-60 min.	- 30 min	
Climbing stairs (daily)	+ 16	4-16	- 4	
At least 30 min of more than moderate intensity (weekly)	+ 3	1-3	- 1	

Weekly consumption	***+1***	***0***	***1***	**+1, 0 or -1**
Vegetables and salads	+ 12	6-12	- 6	
Pieces of fruit	+ 12	6-12	- 6	
Nuts	+ 3	1- 3	- 1	
EVOO	+ 6	3 - 6	- 3	
Oily fish and iberian ham	+ 3	1 - 3	- 1	
Wholegrain cereals	+ 6	3 - 6	- 3	
Legumes	+ 2	1 - 2	- 1	
Skimmed dairy	+ 6	3-6	- 3	
Red and processed meats	- 3	3-6	+ 6	
Sauces (except mayonnaise, ali oli with EVOO)	- 2	2- 4	+ 4	
Sugary drinks (includes juices)	- 2	2 - 4	+ 4	
Cookies and ready-sliced bread	- 2	2- 4	+ 4	

A physician with expertise in clinical and research activities focused on diabetes physiopathology, complications and prevention will be responsible to obtain fasting blood samples to determine HbA1c, lipid profile, γ-tocopherol levels (to confirm compliance in the group receiving pistachios), as well as a first-morning urine sample for the analysis of albumine-to-creatinine ratio and hydroxytyrosol levels (to confirm compliance in the group receiving EVOO). The physician will be also responsible to recollect clinical and lifestyle data from all women.

Prof. Calle-Pascual will manage the global project and will develop a program for institutional relations.

STATISTICAL ANALYSIS

Analysis of all the variables pertaining to efficacy will be performed on an intention-to-treat basis, including data on all patients randomized and all available follow-up data. The Student's t test or ANOVA [nonparametric test (RANK TEST)] will be used to compare continuous variables [and expressed as means standard deviation (SD)] while categorical variables will be compared using the chi-squared test. The association between qualitative variables will be analyzed by the chi-squared test or Fisher's exact test in case more than 25% of the expected values will be less than five. In the case of ordinal variables, the hypothesis of ordinal proportion tendency will be tested. Relative risk will be assessed together with its 95% confidence interval, using Cornfield's method. In all cases, the distribution of the variable versus the theoretical models will be checked, and the variance homogeneity hypothesis will be tested. The paired Student's t test or a repeated measures analysis of variance (MANOVA) will be used to compare continuous variables. The changes in variables with time (pre- and post-treatment) will be estimated by a MANOVA. Variables included will be treatment and study period. Mathematical models will be chosen by the type of variable and used logistic analysis, linear regression or Cox regression. The existence of interactions will be evaluated. Variables showing a p value under 0.15 in the univariate analysis will be selected for the multivariate analyses. The null hypothesis will be rejected in each statistical test when $p < 0.05$. Analysis will be performed using windows SPSS version 17.0 software and STATA 12.

Interim analyses: No applicable.

ETHIC, DEONTOLOGICAL AND REGULATORY CONSIDERATIONS

The Investigator will ensure that this study is conducted in accordance with the principles of the Declaration of Helsinki, ICH Guidelines for Good Clinical Practice and in full conformity with relevant regulations. The protocol, informed consent form, participant information sheet and any applicable documents will be submitted to an appropriate Ethics Committee (EC) and Regulatory Authority for written approval. The trial staff will ensure that the participants' anonymity is maintained. The participants will be identified only by a participant ID number on the CRF and any electronic database. All documents will be stored securely and only accessible by trial staff and authorized personnel. The study will comply with the Data Protection Legislation which requires data to be anonymized as soon as it is mandatory to do so.

ADENDUM, November 30, 2014

- IG will be provided 10 L of EVOO and 2 kgs of pistachios at 12-14 GWs (Visit 1) for free, to ensure compliance to the intervention of at least 12 weeks before attending GDM screening.

- Pistachios are chosen as nuts for IG, because their consumption can decrease appetite and involve glucose- and insulin-lowering effects. Consequently, serum γ-tocopherol, a biomarker of pistachio intake, will be assessed.

- Adherence biomarkers will be measured at baseline and at 24-28 GW in 10% of participants randomly selected from the IG and CG.

- The 14-point Mediterranean Diet Adherence Screener (MEDAS) will be also used to obtain the MEDAS-derived PREDIMED score (**Figure 3**).

- The study will be single-blinded. The staff involved in the trial (doctors, midwifes, evaluator...) might know of the pregnant women's participation in the study, but whether they belong to the CG or IG will remain unknown, excepting to the dieticians. Between 12-14 and 24-28 GWs there will be no visits programmed by the staff of the trial.

Figure 3- MEDAS questionnaire.

**Questions**	**Criteria for 1 point**
1. Do you use olive oil as main culinary fat?	Sí
2. How much olive oil do you consume in a given day (including oil used for frying, salads, out-of-house meals, etc.)?	≥4 tbsp
3. How many vegetable servings do you consume per day? (1 serving : 200 g [consider side dishes as half a serving])	≥2 (≥1 portion raw or as a salad)
4. How many fruit units (including natural fruit juices) do you consume per day?	≥3
5. How many servings of red meat, hamburger, or meat products (ham, sausage, etc.) do you consume per day? (1 serving: 100–150 g)	<1
6. How many servings of butter, margarine, or cream do you consume per day? (1 serving: 12 g)	<1
7. How many sweet or carbonated beverages do you drink per day?	<1
8. How much wine do you drink per week?	≥7 glasses
9. How many servings of legumes do you consume per week? (1 serving : 150 g)	≥3
10. How many servings of fish or shellfish do you consume per week? (1 serving 100–150 g of fish or 4–5 units or 200 g of shellfish)	≥3
11. How many times per week do you consume commercial sweets or pastries (not homemade), such as cakes, cookies, biscuits, or custard?	<3
12. How many servings of nuts (including peanuts) do you consume per week? (1 serving 30 g)	≥3
13. Do you preferentially consume chicken, turkey, or rabbit meat instead of veal, pork, hamburger, or sausage?	Sí

14. How many times per week do you consume vegetables, pasta, rice, or other dishes seasoned with sofrito (sauce made with tomatoand onion, leek, or garlic and simmered with olive oil)?	≥2

Natural fruit juice and alcohol consumption will not be accounted for. Adapted from Martínez-González MA et al. PLoS ONE. 2012; 7(8): e43134.

Final statystical analysis

Sample size

For sample size calculation the primary end-point was the incidence of GDM between 24-28 GWs. We estimated that 315 women per group would be required to provide statistical power of 80% (2-tailed α= 0.05) to detect a relative risk reduction of at least 30% after intervention assuming an incidence of 35% GDM in the control group. Previously, this intervention has shown a decrease of the median of fasting blood glucose (FBG) -0.38 mmol/L after 3 months[21], representing a potential reduction of GDM rate higher than 30%. Taking into account loss to follow-up, we included 1000 successive women that attended their first ultrasound visit at the Obstetrics department between 12-14 GW, to guarantee a minimum duration of 12-weeks of intervention.

Statistical analysis.

Variables are presented with their frequency distribution. Quantitative variables are summarized by their mean and standard deviation (±SD). All primary analyses were performed on an intention-to-treat basis. Comparison between baseline group characteristics and primary and secondary outcomes for qualitative variables were evaluated by the chi-squared test. For quantitative variables, measures were compared through Student's t test or the nonparametric Mann–Whitney U test if the quantitative variables would not adjust to a normal distribution. The Shapiro-Wilk test was used to verify the normal distribution of data.

Logistic regression analyses were used to assess the effect of the intervention for the primary and secondary outcomes that were significantly different in the univariate analysis. The magnitude of association was evaluated using the odds ratio (OR) and 95% confidence interval (CI). A crude and 5 multivariate models were fitted: model 1 was adjusted for age (continuous) ethnicity and parity; model 2 adjusted for pregestational BMI and gestational weight gain (continuous); model 3 adjusted for gestational, personal and family history, and smoker status (never, current, or former smoker); Model 4 adjusted for the variables in Models 1 and 2; and Model 5, adjusted for the variables in Models 1-3. These models 4 and 5 were only fitted for the primary outcome due to the small number of events in many of the secondary outcome variables.

All *p* values are 2-tailed at less than 0.050. Analyses were done using SPSS, version 21 (SPSS, Chicago, Illinois).

ADENDUM April 01, 2017.

Statistical analysis.

At the suggestion of the statistic (M.F.) the crude relative risk has been estimated for each outcome of interest and different methods have been tested for the estimation of adjusted relative risks in cohort studies and clinical trials of common outcomes (1,2,3): a method proposed by Zhan and Yu (2) to correct the adjusted odds ratio in cohort studies of common outcomes and estimate adjusted relative risk and its confidence interval when the incidence of disease is common and adjust for confounding variables exist; the log-binomial model (one of the criticisms of using the log-binomial model for the RR is that it produces confidence intervals that are narrower than they should be, and another is that there can be convergence problems; in our case we have problems of convergence to provide parameter estimates); Poisson regression with robust error variance and Cox regression with robust variance to obtain corrected confidence intervals.

References:

1. McNutt LA, Wu C, Xue X, Hafner JP. Estimating the relative risk in cohort studies and clinical trials of common outcomes. Am J Epidemiol. 2003 May 15;157(10):940-3.
2. J. Zhang and K. Yu, 1998. What's the Relative Risk, JAMA, Vol 280, No 19, pp 1690-1691.
3. Zou G. A Modified Poisson Regression Approach to Prospective Studies with Binary Data. Am J Epidemiol 2004; 159(7):702-6. 3

ADENDUM, May 4, 2017

Follow-up.

All participants were followed-up at the obstetrics department throughout the pregnancy. From the 24-28 GW forward, if women from both the CG and IG were diagnosed with GDM, they were followed-up at the Diabetes and Pregnancy Unit. Both groups of women, regardless of belonging to the CG or IG, were given the same lifestyle recommendations to treat the GDM:

Women were advised to avoid or limit the intake of refined carbohydrates, sugary drinks including fruit juices, red meat (particularly processed meat), bakery and pastries. At least 3 servings of skimmed milk dairy products per day, 2 servings of vegetables per day, and 2 servings of fresh fruit instead of juices per day were recommended. Nuts instead of processed meat snack, oily fish, and virgin olive oil daily and whole grain cereals and legumes instead of white cereals and potatoes were also recommended. A leisure-time physically active lifestyle including walking at least 15 minutes, and climbing stairs at least 4 floors, 4 times a day > 5 days per week, were recommended.

On the other hand, both women with and without GDM diagnosis had a regular obstetric care throughout the pregnancy. This was independent to which group they were randomized to.

Therefore, follow-up by the dietician was done with independence to medical care. It did not overlap with scheduled medical visits. However it did coincide with the scheduled appointments in clinical analysis.

- Control group (CG). Women were assigned to a standard treatment based on recommendations provided in clinical practice. The main recommendation was to reduce all types of fat from both animal and vegetable sources including nuts and EVOO, and a training program is also provided. This prudent diet recommendation represents a contribution of total fat less than 30% of total energy intake (<40 mL EVOO and not nuts consumption), and carbohydrate intake more than 50%. These

recommendations provided are based on national clinical guidelines, and were provided at Visits 1, 2 and 3 by the midwifes.

- **Intervention group (IG).** Women with free allotments of EVOO (1 litre/week) and pistachios (150 g/week), and the training program. Dieticians gave personalized dietary advice to participants with instructions directed to use of EVOO for cooking and dressing, increased consumption of fruit, vegetables, legumes, and fish and avoidance of red or processed meat, butter, cream, fast food, sweets, pastries, and sugar-sweetened beverages. This intervention diet is comprised of an intake of approximately 35-40% of total fat (predominantly unsaturated fatty acids) and 40-45% of low glycemic index carbohydrates, maintaining a protein intake of 20%, similar to the control diet.
The dietician was responsible of giving nutritional advice to women at Visits 1 (12-14GW), 2 (24-28 GW) and 3 (36-38 GW). This was done in order to benefit from the already-programmed visits to the clinical analysis.
