# Supplementary material for: A Mediterranean diet with additional extra virgin olive oil and pistachios reduces the incidence of gestational diabetes mellitus (GDM): A randomized controlled trial: The St. Carlos GDM prevention study
Source: PLoS One. 2017 Oct 19;12(10):e0185873. doi: 10.1371/journal.pone.0185873 (PMC5648128; doi:10.1371/journal.pone.0185873)
Supplement: S3 File — Spanish version. (PDF) [file pone.0185873.s003.pdf]

Reducción de la aparición de Diabetes Gestacional por adherencia a la dieta mediterránea

TITULO: Reducción de la aparición de Diabetes Gestacional por adherencia a la dieta mediterránea

INVESTIGADOR/A PRINCIPAL:ALFONSO LUIS CALLE PASCUAL

RESUMEN (Objetivos y Metodología del Proyecto) (Máximo 250 palabras)

Objetivo Definir la tasa reducción de aparición de DMG en la semana 24-28 según los criterios HAPO tras una intervención con dieta mediterránea y ejercicio físico comparada con el tratamiento habitual en mujeres gestantes que han tenido unos niveles de glucosa en ayunas normales (<92 mg/dL) en la primera visita gestacional entre las semanas 8-12. Conocer los efectos de la intervención en: - biomarcadores inflamatorios -mecanismos epigenéticos (DNA metilación y expresión miRNA) - cambios en HbA1c, insulina, HOMA IR, perfil lipídico, tensión arterial, peso corporal La Duración de la gestación, hipetension inducida por la gestación, partos preterminos (<37 semanas), desarrollo fetal ecográfico (diámetro abdominal, torácico, biparietal y longitud femur),características del parto, peso del recién nacido, valor Apgar, ph cordon y morbilidad perinatal

Diseño del estudio: Unicentrico prospectivo randomizado de intervencion con 2 grupos paralelos

Intervención: Las mujeres elegibles serán asignadas a GRUPO CONTROL, con seguimiento y tratamiento usual por los obstetras ó al GRUPO MedDiet, donde recibirán 1l de aceite de oliva virgen extra y 150 g frutos secos para cada 2 semanas para garantizar la adherencia al tratamiento nutricional propuesto por la nutricionista con el objetivo de alcanzar una puntuación >10 segun cuestionario previamente validado, basado en el estudio Diabetes Nutrition and Complications trial (DNCT) previamente publicado

JUSTIFICACION

Varios factores del riesgo modificables para la aparición de la Diabetes tipo 2 se han identificado en mujeres (1), y la Dieta del Mediterráneo (MedDiet), ha demostrado ser eficaz para prevenir su aparición (2-3). La Diabetes Gestational (GDM) es un problema de la salud creciente a causa de su prevalencia creciente y sus efectos adversos sobre la madre, la gestación y el recien nacido (4) y aumenta el riesgo postnatal para el desarrollo de diabetes tipo 2 y enfermedad cardiovascular (5). La aplicación de los nuevos criterios para diagnosticar GDM, después del estudio HAPO (6), se ha asociado a un incremento en la prevalencia de GDM del 8% (NDDG criterios) y 10,6% (Carpenter Coustan) hasta el 35.5% en nuestra población de mujeres (7-8), como recientemente hemos publicado. A pesar de que los puntos de corte de glucemia (categoria 5) son mas estrictos que los utilizados con anterioridad, El estudio HAPO muestra una asociación continua entre los niveles de glucosa maternales y los niveles de péptido C en el cordon umbilical y los eventos adversos abajo esos previamente usaron a GDM diagnosis con aumentó peso del nacimiento y resultados del embarazo adversos. Con respecto a este aumento dramático en la prevalencia de GDM con los criterios propuestos por el IADPSG, recientemente nuestro grupo (8) ha demostrado que a pesar del incremento en la

prevalencia de Diabetes Gestacional (x 3,5), la misma intervención recomendada produce un descenso en la tasa de hipertensión inducida por la gestación (4.1 -3.5%: -14.6%, $p < 0.021$), prematuridad (6.4 -5.7%: -10.9%, $p < 0.039$), cesareas (25.4 -19.7%: -23.9%, $p < 0.002$), recién nacidos pequeños para edad gestacional (7.7 -7.1%: -6.5%, $p < 0.042$), grandes para edad gestacional (4.6 -3.7%: -20%, $p < 0.004$), recién nacidos con Apgar al minuto <7 (3.8 -3.5%: -9%, $p < 0.015$), and admisión en UCI neonatal (8.2 -6.2%: -24.4%, $p < 0.001$). Considerando la mejoría en los objetivos de salud, de la mujer y del recién nacido induce un ahorro estimado basado en los GRDs de mas de 14.000 € por cada 100 mujeres cribadas y asistidas como se propone. Esto supuso un ahorro de más de 300.000€ en el año 2013 en nuestro área sanitaria. Estos resultados se pueden considerar esperables porque se ha demostrado que mujeres quienes tenían un valor de la prueba anormal y quienes se consideraban previamente como "normales" tenían muchos de los resultados similares a las que estaban diagnosticadas como GDM. Poder reducir su aparición resulta crucial para el sistema sanitario público Recientemente, basado en una población de más de 2000 mujeres identificadas por los anteriores criterios, nuestro grupo ha desarrollado un modelo predictivo pregestacional del riesgo para desarrollar GDM, identificando los siguientes patrones de alimentación modificables que pueden ayudar a predecir el riesgo de GDM (7). Después de regresión logistica, el consumo de galletas y/o pan de molde < 4 veces/ semana, carnes grasas y/o procesadas < 6 raciones/ semana, zumos y bebidas azucaradas < 4 porciones/ semana,y caminar > 30 minutos/ día y realizar al menos 30 minutos/ día de actividad física de intensidad al menos moderada por lo menos 2 días/ se asoció a una reducción en el riesgo para desarrollar GDM en comparación con el consumo opuesto. Este datos representarían una proximidad prometedora por la prevención de GDM y complicaciones subsecuentes. En España la Comunidad de Madrid es una de las regiones con mayor tasa de inmigrantes, 18,8% de la población total, con 49,7% de mujeres. Aproximadamente 50% de mujeres embarazadas asistió a en nuestro hospital durante 2012-2013 eran inmigrantes. Recientemente, hemos publicado la población inmigrante adquiere los mismos factores del riesgo modificables que la población española (9). La obesidad y sobrepeso pregestacional, la edad avanzada y la paridad representan los mas importantes factores de riesgo para desarrollas GDM e incrementa la morbilidad materna y neonatal (9). Lamentablemente tan solo menos de un 10% de las mujeres acuden a consulta preconcepcional para planificar su gestación, y tan solo de forma habitual se prescribe ac folico y suplementos de iodo, pero no se evalua el riesgo para desarrollar GDM. Por ello tiene mucho interés determinar los factores de riesgo modificables asociados a estilo de vida que pueden afectar a la aparición de GDM desde la primera vista prenatal habitualmente entre las semanas 8-10 de gestación. Recientemente ha sido realizado un metaanális sobre las posibles intervenciones sobre el estilo de vida durante es periodo de tiempo entre las semanas 8-10 y 24-28 cuando se realiza el cribaje y diagnóstico de GDM (10-11), sugiriendo que intervenciones sobre la alimentación y actividad física pueden ser efectivas durante este periodo de la gestación. Nuestro grupo, analizando la serie de 1546 mujeres sometidas al cribaje de GDM durante 1 año (8) publicado recientemente, ha identificado 4 patrones de alimentación desde la primera vista concepcional asociados a la aparición de GDM en la semana 24-28. Los riesgos relativos de consumir [illegible]3 veces/semana frutos secos (1.68; IC95%: 1.10-2.55, $p<0.01$), consumir > 1 día/semana cereales refinados (1.39; IC 95%:1.12-1.72, $p<0.005$), consumir 4 días por semana zumos/bebidas azucaradas (1.30; IC95% 1.05-1.62, $p <0.02$) y 4 días por semana galletas/pan de molde/bollos (1.41; IC 95%:1.13-1.76, p

<0.003), así como los de ganar más de 9 ó menos de 5 Kg de peso durante este periodo (1.40; IC 95%:1.13-1.75, p <0.003), han sido identificados en nuestra serie y asociados al desarrollo de GDM. El tener los 4 patrones detectados en bajo riesgo en comparación con alto riesgo despues de una análisis de regresión multiple ajustado por edad, IMC y paridad se asocia a una reducción en la aparición de GDM (OR 0.206 IC 95%: 0.068-0.623, p <0.005). Si hallamos la ROC incluyendo los factores no modificables, antecedentes obstétricos y familiares tiene una sensibilidad de 0.593 una especificidad de 0.666 y una area bajo la curva de 0.657 (0.625-0.689) con una p <0.0001 (Anexo 1). Hasta nuestro conocimiento estos datos pendientes de publicar, son los primeros en la literatura e identifica potenciales patrones de alimentación sobre los que intervenir, y permite desarrollar estrategias preventivas para reducir su aparición, que es lo que pretendemos alcanzar en este proyecto. Las adipoquinas y mediadores inflamatorios son considerados los principales factores que causan inflamación subclínica que tiende a inducir resistencia a la insulina y anormalidad en el metabolismo de la glucosa. Además las evidencias que demuestran que la exposición fetal a un ambiente hostil, como la hiperglucemia materna, pueden influir en la aparición de enfermedades crónicas en un futuro están aumentando (12-15). Así la exposición a un ambiente intrauterino hiperglucémico, junto con susceptibilidad genética puede dar lugar a la aparición de determinadas enfermedades metabólicas y cardiovasculares. Por lo tanto es muy relevante identificar los mecanismos exactos por los que un exceso de glucosa materna puede dar lugar a enfermedades en el recién nacido y puede permitir desarrollar estrategias para prevenir este destructivo ciclo de disfunción metabólica a través de generaciones. En resumen la DMG puede incluir unas series de fenómenos metabólicos, inflamación, modificaciones epigenéticas, metilación y miRNA que pudieran ser modificados por la alimentación (16-19). En este proyecto vamos a investigar el impacto de la dieta mediterránea in la prevención de la DMG : Si la hipótesis es demostrada, dispondremos de muestras para evaluar su relación con estos factores. El análisis simultaneo de estos parámetros puede suministrar nuevos biomarcadores que pueden tener aplicación en el diagnostico y pronostico de la DMG.

1. Hu FB et al. Diet life style and the risk of type 2 diabetes mellitus in women. N Engl J Med 2001;345:790-7

2. Salas-Salvadó J et al. Reduction in the incidence of type 2 diabetes with the Mediterranean diet: results of the PREDIMED-Reus nutrition intervention randomized trial. Diabetes Care 2011;34:14-9

3. The InterAct Consortium et al. Mediterranean Diet and Type2 Diabetes Risk in the European Prospective Investigation into Cancer and Nutrition (EPIC) Study: the InterAct project. Diabetes Care 2011;34:1913-8

4. Casey BM et al. Pregnancy outcomes in women with gestational diabetes compared with the general obstetric population. Obstet Gynecol 1997;90: 869-73

5. Kim C et al. Gestational diabetes and the incidence of type 2 diabetes: A systematic review. Diabetes Care 2002;25:1862-8

6. The HAPO Study Group. Hyperglycemia and Adverse Pregnancy Outcomes N Engl J Med 2008;358:1991-2002

7. Ramos-Levi A et al. Risk factors for gestational diabetes mellitus in a large population of women living in Spain: Implications for preventative strategies. Int J Endocrinol 2012; 312529

8. Duran A et al The introduction of IADPSG criteria for the screnning and diagnosis of Gestational Diabetes Mellitus results in improved pregnancy outcomes at a lower cost in a large cohort of pregnant women: The St Carlos Gestational Study. Diabetes Care 2014 **Ahead of Print, published online June 19, 2014** (DOI: 10.2337/dc14-0179)

9. Pérez-Ferre N et al. Effect of lifestyle on the risk of gestational diabetes and obstetric outcomes in immigrant Hispanic women living in Spain. J Diabetes 2012;4:432-8

10. Radesky JS et al. Diet during early pregnancy and development of gestational diabetes. *Paediatr Perinat Epidemiol* 2008; 22: 47–59. doi:10.1111/j.1365-3016.2007.00899.x.

11. Agha M et al. Interventions to reduce and prevent obesity in pre-conceptual and pregnant women: A systematic review and Meta-Analyssis.PLoS ONE 9(5): e95132. Doi:10.1371/journal.pone.0095132

12.. Guelinckx I et al. Maternal obesity: pregnancy complications, gestational weight gain and nutrition. Obes Rev 2008;9:140-50

13. Vrachnis N et al. Role of adipokines and other inflammatory mediators in gestational diabetes mellitus and previous gestational diabetes mellitus. Int J Endocrinol 2012:549748

14. Lillycrop KA et al. Epigenetic mechanisms linking early nutrition to long term health. Best Pract Res Clin Endocrinol Metab 2012;26:667-76

15. Jones RH et al. Fetal programming of glucose-insulin metabolism. Mol Cell Endocrinol 2009;297:4-9

16: Houde AA et al. Fetal epigenetic programming of adipokines. Adipocyte 2013;2:41-6

17. Palmer JD et al. MicroRNA expression altered by diet: Can food be medicinal? Ageing Res Rev 2014 (doi.org/10.1016/j.arr.2014.04005

18. Morales Prieto DM. MicroRNAs in pregnancy. J Reprod Immunol 2011;88:106-11

19. García-Segura L et al. The emerging role of MicroRNAs in the regulation of gene expression by nutrients. J Nutrigenet Nutrigenomics 2013;6:16-31

HIPÓTESIS

Tradicionalmente esta aceptado que factores genéticos junto con factores asociados a estilo de vida influyen en el desarrollo de algunas enfermedades como la obesidad, la diabetes y la enfermedad cardiovascular en el adulto. Existe suficiente evidencia que sustenta la importancia del ambiente intrauterino en la susceptibilidad a padecer determinadas enfermedades en el futuro pero los mecanismos por los que actúa permanecen por conocer.

Reducción de la aparición de Diabetes Gestacional por adherencia a la dieta mediterránea

Nuestro trabajo quiere probar la hipótesis que una intervención sobre el estilo de vida basada en la dieta mediterránea y el ejercicio físico, que comience en la primera visita gestacional, en mujeres con niveles glucosa en ayunas normales (<92 mg/dl) entre las semanas 8-12 y mantenida durante la gestación puede reducir la tasa de aparición de DMG entre las semanas 24 y 28 y reducir la morbilidad de la madre, la gestación y el recien nacido

OBJETIVOS

Primario: Definir la tasa reducción de aparición de DMG en la semana 24-28 según los criterios HAPO tras una intervención con dieta mediterránea y ejercicio físico comparada con el tratamiento habitual en mujeres gestantes que han tenido unos niveles de glucosa en ayunas normales (<92 mg/dL) en la primera visita gestacional entre las semanas 8-12.

Secundarios: Conocer los efectos de la intervención en:

- biomarcadores inflamatorios

- cambios en HbA1c, insulina, HOMA IR, perfil lipídico, tensión arterial, peso corporal

- Duración de la gestación, hipetension inducida por la gestación, partos preterminos (<37 semanas), desarrollo fetal ecográfico (diámetro abdominal, torácico, biparietal y longitud femur),características del parto, peso del recién nacido, valor Apgar, ph cordon y morbilidad perinatal

- Disponer de muestras para evaluar potencial mecanismos de acción de la intervención desarrollada: -mecanismos epigenéticos (DNA metilación) y expresión miRNA

Diseño, sujetos de estudio, variables, recogida y análisis de datos y limitaciones del estudio.

Las estrategias para la prevención de la DMG están enfocadas hacia la intervención con cambios en estilo de vida favoreciendo la realización de actividad física y mayor adherencia a una alimentación saludable. Recientemente el estudio PREDIMED ha demostrado con una intervención semejante a diseñada en el trabajo actual, una reducción media en los valores de glucemia en ayunas de 7 mg/dl en una población de alto riesgo tras 3 meses de intervención. Si consideramos una prevalencia de DMG del 35.5%, podemos considerar a todas las mujeres gestantes como de alto riesgo. Además podemos también considerar que la gestación es un periodo en el cual se alcanza el mayor grado de adherencia a los tratamientos propuestos. Si se reprodujeran los resultados esperables, se produciría una reducción mayor del 40% de la aparición de DMG, ya que el 55% de los casos se diagnostican por glucemia basal en ayunas entre 92 y 99 mg/dl. Para evaluar si es posible reducir la aparición de DMG se ha diseñado un estudio de intervención, prospectivo y aleatorizado,unicentro, con 2 grupos paralelos. El estudio ha sido aprobado por el Comité Ético del hospital Clínico de San Carlos (17/07/2013, acta 7.2/13, se adjunta documento) y ha sido registrado (ISRCTN84389045 http://www.controlled-trials.com/ ISRCTN84389045)

Reducción de la aparición de Diabetes Gestacional por adherencia a la dieta mediterránea

Tamaño Muestral: Para la estimación del tamaño muestral se considera una tasa de conversión a DMG de al menos 35% entre las semanas 24 y 28 de gestación y una reducción con la intervención >30%. Un tamaño muestral de 315 mujeres por grupo suministra al estudio una potencia >80% para detectar >30% de diferencia en las tasas de conversión entre los grupos con un 5% de significado. Teniendo en cuenta, rechazo a la participación, y pérdidas durante el seguimiento, se van a invitar a participar a las 1000 mujeres sucesivas que acudan a la primera visita prenatal al servicio de Obstetricia entre las semanas 8-12 de gestación para garantizar una duración mínima de 3 meses de intervención. (Anexo)figura 1 Deberá cumplir los criterios de inclusión y exclusión y firmar el consentimiento informado.

Criterios de inclusión: Todas las mujeres gestantes mayores de 18 años de edad con niveles de glucosa en ayunas <92 mg/dl en la primera consulta prenatal (entre la semanas 8-12 de la gestación) serán invitadas a participar en el estudio y firmaran un consentimiento informado si aceptan participar.

Criterios de exclusión: Mujeres gestantes con niveles de glucosa en ayunas > 92 mg/dl en la primera valoración prenatal, intolerancia a frutos secos/aceite de oliva, gestación múltiple, y cualquier enfermedad médica ó tratamientos farmacológicos que a juicio del equipo investigador pueda alterar los efectos de la intervención, ó estén sujetas a tratamiento nutricionales específicos. Intervención: Las mujeres elegibles serán aleatorizadas, considerando su edad, origen étnico, peso corporal pregestacional y paridad, en 2 grupos, intervención y control.

Grupo Control: Las mujeres asignadas al grupo Control, seguirán unas recomendaciones habituales durante la gestación, y seguidas como habitualmente por el servicio de obstetricia. Las recomendaciones habituales actuales contemplan unas indicaciones para reducir el consumo de grasa total (<30%) limitando el consumo de aceite de oliva y frutos secos, embutidos y carnes grasas, mantener el de carbohidratos sobre un 50% y proteínas 20%.

Grupo Intervención MedDiet: Las mujeres asignadas al grupo de intervención recibirán educación nutricional suministrada por una Nutricionista, favoreciendo el consumo de grasa mono-insaturada y poli-insaturada, sobre todo en ensaladas aderezadas con aceite de oliva, y acompañada habitualmente de frutos secos, y pescado azul pequeños (sardinillas, caballas ó melva) que según análisis propios contienen baja cantidad de metil mercurio y se pueden consumir con seguridad en poblaciones vulnerables como las gestantes. Se sumistrará 1 litro de aceite oliva virgen extra y 150 gramos de frutos secos por cada 2 semanas. De forma simultanea, se recomendará incrementar el consumo de fruta entera en lugar de zumos, cereales integrales en lugar de blancos, evitando el consumo de galletas, productos de bollería industrial, mermeladas y pan de molde y alimentos precocinados industriales. Esta intervención contiene aproximadamente entre 35-40% de grasa, siendo >50% monoinsaturada y poliinsaturada, entre 40-45% de carbohidratos con baja carga glucémica y bajo índice glucémico, y manteniendo un consumo de alimentos ricos en proteínas sobre el 20% de las calorías totales, semejante al grupo control. El objetivo es alcanzar una puntuación en el cuestionario de adherencia a dieta mediterránea >10 puntos. Las mujeres recibirán un litro de aceite de oliva virgen extra y 150 gramos de frutos secos cada 2 semanas. Las mujeres del

grupo de intervención serán seguidas por el Servicio de Endocrinología conjuntamente con el servicio de Obstetricia.

Aleatorización: Se construirá una matriz de aleatorización por bloques de permutación, estratificadas por edad, IMC paridad y origen étnico y asignadas a cada grupo (1:1) en bloques de 4-6 (Anexo Figura)

Seguimiento: Se programan las siguientes visitas:

Visita 0, entre 8-12 semanas gestación: Firma del consentimiento, historia clínica, muestra sangre y cuestionario de alimentación.

Visita 1: una semana mas tarde, cumplimiento de criterios de inclusión, aleatorización, obtención de muestra de sangre y suministro de la intervención.

Visita 2: entre las semanas 16-18, cuestionario de nutrición, adherencia a las recomendaciones, seguimiento médico/educativo

Visita 3: Entre las semana 24-28, garantizando al menos 12 semanas de intervención, con la sobrecarga oral con 75 gramos de glucosa, identificación de DMG basado en los criterios IADPSG, aplicación de cuestionario de alimentación y obtención de muestra de sangre y orina.

Visitas 4-5(y 5 bis, según sean necesarias): Semanas de gestación 28 antes38 según estén identificadas de DMG, para seguimiento de la DMG

Visita 6: En la semana de gestación 38, para evaluación de adherencia, cuestionario alimentación y obtención de muestra de sangre y orina.

Vista 7: A los 3 meses postparto. Evaluación clínica, cuestionario de alimentación y muestra de sangre. Determinación de biomarcadores: De las muestras sanguíneas/orina, obtenidas para este estudio se determinarán:

- Parámetros de adherencia a la intervención: Niveles de hidroxitirosol urinario y ácido alfa linoleico plasmático y cuestionario de alimentación.

- Parámetros de eficacia de la intervención: Cambios en niveles de glucémia basal y tras SOG, tasa de conversión a DMG, insulina en ayunas, HOMA, perfil lipoproteínas, adiponectina, y Vitamina D.

- Parámetros antropométricos Peso corporal y tensión arterial

- Parámetros de la gestación: Duración de la gestación, y aparición de complicaciones, en particular hipertensión arterial y eclampsia

- Parámetros fetales: Desarrollo ecográfico con estimación de los perímetros abdominal y biparietal, longitud de femur, crecimiento y desarrollo por percentiles, estimación del peso. Sufrimiento y perdida del bienestar fetal

- Parámetros del parto: Tasas de cesáreas, partos instrumentales y complicaciones.

- Parámetros del recién nacido: Peso, test Apgar, ph del cordón, tasas de síndrome de hijo de madre diabética, necesidad de ingreso en cuidados intensivos neonatologicos y sus causas

Muestras para poder determinar si se demuestra la hipótesis, y con cargo a otros fondos posibles: Leptina, hsCPR, cCD40L, Lp-PLA2, EPCs circulantes ((CD34+; CD34+/CD144 (vecadherin)+; CD14+/CD16+ , análisis de metilación del DNA y aislamiento y cuantificación de miRNA

Reclutamiento de pacientes: 18 meses: Entre Enero-2015 Junio 2016. Participarán los facultativos especialistas de área de obstetricia (Dra Nuria Izquierdo y Dra Noelia Pérez) del Hospital Clínico San Carlos (CEP, Modesto Lafuente y Av Portugal). Intervención y Seguimiento de las mujeres seleccionadas: Enero 2015- Marzo 2017. La aleatorización será responsabilidad Dr A.L. Calle-Pascual El grupo Control será seguido por los obstetras de forma habitual (Dra N Izquierdo y Dra N Pérez). El Grupo de Intervención MetDiet será educado y seguido por la nutricionista contratada con cargo al proyecto, quien aplicará el cuestionario de alimentación, y realizará la intervención nutricional y actividad física, suministrando el aceite de oliva virgen extra (1l/cada 2 semanas) y 150 gramos de frutos secos, que se consumirán acompañando a las ensaladas y como medias mañanas y/o meriendas en lugar de embutidos blancos y bocadillos/galletas/snack, recomendando la realización de actividad física habitual. El seguimiento médico con la obtención de datos antropométricos, tensión arterial y datos clínicos se realizar en los CEP del Hospital Clínico San Carlos, por los Drs A.L. Calle Pascual, Cristina Familiar, N. Pérez-Ferré, Mª Paz de Miguel y A. Durán Recolección de los datos clínicos y bioquímicos: Enero 2015-Diciembre 2017. Las muestras de sangre y orina se obtendrán en el Laboratorio del Servicio de Endocrinología del Hospital Clínico San Carlos por la enfermería responsable y manipuladas por la Dra Bordiú. Los parámetros bioquímicos generales (glucosa/HbA1c/Insulina/ lipidos/ adiponectina/ Vitamina D) serán evaluados en cada visita (Laboratorio Endocrinología).

Obtención de las muestras para este estudio que se solicita subvención: Enero 2015-Diciembre 2017. Obtención de las muestras de suero: Se recogerán las muestras de sangre en dos tubos de suero con gel separador tras una noche de ayuno. Se dejarán los tubos a temperatura ambiente (TA) 1 h para favorecer la retracción del coágulo. Se centrifugarán los tubos 10 min a 1300g a TA . Se transferirá la fase superior en un tubo nuevo para realizar una segunda centrifugación de 5 min a 3000g a TA. Se transferirá cuidadosamente el sobrenadante a diferentes alícuotas sin tocar el pellet y se almacenarán a -80 ºC hasta su procesamiento. Extracción de miRNAs de suero:Se realizará con el kit de aislamiento Qiagen miRNeasy Mini Kit, añadiendo RNA de bacteriófago MS2 y RNA spike-in mix (UniSp2, UniSp4 and UniSp5). El RNA extraído se almacenará a -80 ºC. Se realizará una extracción de ADN genómico,se almacenará a -80º,a partir de muestras de sangre periférica en todas las pacientes en el momento de confirmación del embarazo y posteriormente en el momento del screening de diabetes gestacional asi como de sangre del cordón fetal. La sangre se recogerá en tubos de 10mL con EDTA. La purificación del ADN se realizará mediante el kit de extracción con DNAzol® - Genomic DNA Isolation Reagent (Molecular Research Center, Inc., Cincinnati, OH) para 2.100 muestras.

Reducción de la aparición de Diabetes Gestacional por adherencia a la dieta mediterránea

Los niveles de hidroxitirosol en orina para la ingesta del aceite de oliva extra virgen, por cromatografía de gasesespectrometría de masas y los niveles de alfa-linolénico en plasma para la ingesta de los frutos secos, por cromatografía de gases. Ambos marcadores se analizarán fuera del hospital, en la facultad de Químicas UCM (contratación externa).Explotación de los Datos: Tasa de reducción en la aparición de Diabetes Gestacional Octubre de 2016. Evolución de la Gestación, Diciembre de 2016. Evaluación Postparto Junio 2017. Supervision del proyecto, recoleccion de Datos y muestras y Evaluacion interna, Dra Inmaculada Moraga supervisará a los MIR 3 de nuestro servicio (A. Ortolá y I Crespo). Este proyecto puede significar una oportunidad para la iniciación de la investigación para los MIR y la realización de su tesis doctoral.

Anexo 1: Curva ROC, sensibilidad y especificidad para identificar las mujeres con DMG, incluyendo los 4 patrones de alimentación detectados en el análisis univariado como variable categórica considerando consumo en riesgo(0; 1-2; 3-4); BMI (<25; >25); Edad (<35; >35); Antecedentes Obstetricos (ninguno; al menos 1); Antecedentes Familiares (Ninduno; al menos un componente del S Metabólico); Actividad física (sedentario, moderamente activo):

Sensibilidad (0.593) Especificidad (0.666) y Area baljo la curva ROC (0.657) (IC 95%:0.625-0.689; p <0.0001)

El alcanzar los 4 patrones de alimentación detectados en bajo riesgo:>3 veces/semana frutos secos; < 1 día/ semana cereales refinados, <4 días por semana zumos/bebidas azucaradas y <4 días por semana galletas/ pan de molde/bollos, representa la base de la intervención, e intentar evitar ganar más de 9 ó menos de 5 Kg de peso hasta la semana 24-28 de gestación, puede reducir la aparición de la DMG, basado en datos previos.

Obviamente se considera imprescindible garantizar la ingesta de 4 raciones de lácteos descremados, y los suplementos de iodo y vitaminas necesarias durante la gestación

Anexo 2: Cuestionario de Seguimiento

Actividad Física

	***A* (+1)**	***B* (0)**	***C* (-1)**
Pasea al día	Mas de 1 hora	30-60 minutos	Menos de 30
Subo pisos por día	Mas de 16	Entre 4 y16	Menos de 4
>30´ de deporte moderado (por semana)	Mas de 3 dias	Entre 1 y 3	Menos de 1

número de veces que consume/ por semana	***A* (+1)**	***B* (0)**	***C* (-1)**
Verduras y/o ensaladas	Mas de 12 veces.	Entre 6 y 12	Menos de 6
Piezas de Frutas	Mas de 12 piezas	Entre 6 y 12	Menos de 6

Frutos secos	Mas de 3 días Entre 1 y 3 Menos de 1
Aceite de oliva virgen	Mas 6 días Entre 3 y 6 Menos de 3
Pescado Azul	Mas de 3 días Entre 1 y 3 Menos de 1
Pescado Blanco	Mas de 3 días Entre 1 y 3 Menos de 1
cereales **integrales**	Mas de 6 días Entre 3 y 6 Menos de 3
Arroz/Pan Blanco	Menos de 3 días Entre 3 y 6 Mas de 6
Legumbres	Mas de 2 días Entre 1 y 2 Menos de 1
Lácteos desnatados	Mas de 6 días Entre 3 y 6 Menos de 3
Carne grasa	Menos de 3 días Entre 3 y 6 Mas de 6
Carnes procesadas	Menos de 2 días Entre 2-3 Mas de 3
Salsas mostaza o similares	Menos de 2 días Entre 2 y 4 Mas de 4
Bebidas azucaradas	Menos de 2 días Entre 2 y 4 Mas de 4
Galletas/Pan Precocinados	Menos de 2 días Entre 2 y 4 Mas de 4
Café (de cualquier tipo)	Mas de 3 cada día Menos de 3
Agua con comidas	Exclusiva Mixta Nunca
